# Evolving Strategies for Producing Multiscale Graphene‐Enhanced Fiber‐Reinforced Polymer Composites for Smart Structural Applications

**DOI:** 10.1002/advs.201903501

**Published:** 2020-04-08

**Authors:** Azadeh Mirabedini, Andrew Ang, Mostafa Nikzad, Bronwyn Fox, Kin‐Tak Lau, Nishar Hameed

**Affiliations:** ^1^ Faculty of Science, Engineering and Technology Swinburne University of Technology Hawthorn VIC 3122 Australia; ^2^ DMTC Limited (Australia) Hawthorn VIC 3122 Australia

**Keywords:** composites, graphene, polymers, smart materials

## Abstract

Graphene has become an important research focus in many current fields of science including composite manufacturing. Developmental work in the field of graphene‐enhanced composites has revealed several functional and structural characteristics that promise great benefits for their use in a broad range of applications. There has been much interest in the production of multiscale high‐performance, lightweight, yet robust, multifunctional graphene‐enhanced fiber‐reinforced polymer (gFRP) composites. Although there are many reports that document performance enhancement in materials through the inclusion of graphene nanomaterials into a matrix, or its integration onto the reinforcing fiber component, only a few graphene‐based products have actually made the transition to the marketplace. The primary focus of this work concerns the structural gFRPs and discussion on the corresponding manufacturing methodologies for the effective incorporation of graphene into these systems. Another important aspect of this work is to present recent results and highlight the excellent functional and structural properties of the resulting gFRP materials with a view to their future applications. Development of clear standards for the assessment of graphene material properties, improvement of existing materials and scalable manufacturing technologies, and specific regulations concerning human health and environmental safety are key factors to accelerate the successful commercialization of gFRPs.

## Graphical abstract

This work considers the key approaches for the introduction of graphene nanomaterials into fiber‐reinforced polymer composites so‐called nanoaugmentation and nanoengineering methods to produce multiscale smart composites. The ultimate goal of this work is to facilitate the faster industrial adoption of graphene products by filling current barriers that the technology faces, including supply chain challenges, lack of safety and standardization regulations, as well as the research gaps.

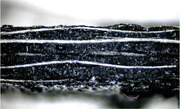

## 1 Introduction

With the ever‐increasing demand for the replacement of conventional structural materials with lightweight functional alternatives, advanced composite structures have, justifiably, drawn increasing attention from the research community. State‐of‐the‐art developments in the field of composite materials have shown outstanding potential for their use in a wide variety of devices and products ranging from large aerospace components all the way to small consumer goods.^[^ ^1^, ^2^ ^]^ Fiber‐reinforced polymer (FRP) composites, introduced years ago, are among the most frequently employed components for many structural applications due to their light weight, improved mechanical properties, chemical stability, versatile processing techniques and controlled corrosion and fatigue.^[^ ^3^, ^4^, ^5^ ^]^ Glass fiber (GFs) and carbon fibers (CFs) are two of the common reinforcing fibers used in conventional polymer composites. However, conventional FRPs suffer from a number of common issues caused by a mismatch of physical and interfacial properties between binding polymer matrices and fabric components. Moderate improvement in mechanical strength is countered by reduced toughness and susceptibility to impact and catastrophic sudden failure caused by weak interfacial bonding and presence of interfacial defects. Rapid degradation arises from differences in the thermal expansion coefficients of matrix and fibers and significant manufacturing costs may be a further limitation.^[^ ^5^ ^]^

Nanoscale reinforcing fillers have emerged as a promising new class of materials for the development of reinforced composites which benefit from increased specific interfacial area, controlled interfacial interactions and enhanced overall compliance.^[^ ^6^, ^7^ ^]^ With this in mind, a variety of carbonaceous nanomaterials has been proposed and employed individually or in hybrid forms. Among these, carbon nanotubes (CNTs) and graphene have been widely explored for their incorporation into polymer composites, due to their combined unique physical and chemical properties, to develop functional materials.^[^ ^8^, ^9^ ^]^ Early investigations have shown that while CNTs may enable higher mechanical, electrical, and thermal properties than graphene,^[^ ^10^ ^]^ in the case of polymer composite materials, their low yield production, relatively high production costs and complex processing hinder their incorporation in large‐scale practical applications.^[^ ^11^ ^]^ Graphene has drawn much attention as a prospective carbon‐based nanomaterial since it was first prepared by the micromechanical cleavage of graphite in 2004.^[^ ^8^, ^12^, ^13^ ^]^ The 2D honeycomb crystal lattice of sp^2^‐hybridized carbon which exists as a single atom thickness,^[^ ^14^ ^]^ has recently developed a great deal of industrial interest in the field of “smart” composite materials due to its unique combination of mechanical, thermal and electrical properties which make it one of the key enablers for high‐performance smart composite applications.^[^ ^15^, ^16^, ^17^, ^18^, ^19^ ^]^

A critical point for nanofillers is known as the percolation threshold; the point at which the filler forms a connected network. One can observe a drastic increase in conductivity of the overall composite around this critical filler concentration point.^[^ ^20^ ^]^ Thus, the composite essentially goes from being mostly insulating to conductive as the current can now flow through the conductive network without being insulated by the surrounding matrix. The percolation threshold purely governed by the filler size and the geometry which is often characterized by its aspect ratio, also known as a length‐to‐diameter ratio.^[^ ^21^ ^]^ Graphene's flat sheet microstructure means that it has an extremely low aspect ratio (often less than 0.01), while the long cylinder microstructure of CNT leaves it with a large aspect ratio (often larger than 100). Both extreme values produce a low percolation threshold. The electronic properties of graphene are found to be crucially dependent on the geometrical shape and chemistry of its edge boundaries. Several experimental studies have indicated the dramatic influence of the crystallographic orientation of graphene 1D edges (i.e., zigzag and armchair) on the electronic structure of graphene.^[^ ^22^ ^]^ The electronic properties of graphene edges are also significantly affected by how foreign chemical species bond to the edge carbon atoms.^[^ ^23^ ^]^ It is therefore considered as an important aspect in the fabrication of functional graphene‐based composites to tune the electronic, magnetic and chemical properties of graphene‐based nanomaterials by changing the chemistry and geometrical shape of the graphene edge.

The recent introduction of graphene‐based nanomaterials into FRP composites has led to many significant improvements in structural and nonstructural functionalities and prompted the development of a variety of smart composites for emerging novel applications. Combinations of micro–nano filler types may prove advantageous in the final product since the ultimate properties can be a result of additive or synergistic effects between the fillers.^[^ ^24^ ^]^ Moreover, the hybridization procedure used can counterbalance some of the disadvantages of a specific reinforcing filler, as it may enhance interactions within the matrix depending on the functionalization route.^[^ ^25^ ^]^ Graphene nanomaterials have been initially employed in FRPs to enhance structural properties, including stiffness and strength, alongside other mechanical characteristics such as fracture toughness, energy absorption and thermal stability. Graphene, known as an “active smart material,” has also become an attractive component for production of high‐performance “stimuli‐responsive” or “self‐sensing” composites.^[^ ^26^ ^]^ Functional properties, such as electrical and thermal conductivity, sensing and monitoring, actuation, energy harvesting/storage, self‐healing capability, electromagnetic interference (EMI) shielding, recyclability, and biodegradability have also been shown to be significantly improved through the proper integration of graphene materials into FRP composites.^[^ ^27^ ^]^ One critical barrier that remains, however, to the industrial acceptance of these materials, is how to achieve tangible material improvements incorporating graphene nanospecies into the final composite structures with minimal changes in the existing manufacturing methods and infrastructure.

Many advances have been made over the last decade in our fundamental understanding of graphene modified polymer composites. Yet, to date, multiscale graphene‐enhanced fiber‐reinforced polymers (gFRPs) lack an inclusive published review that specifically summarizes their emergence, technologies of processing and fabrication, as well as their characterization and modelling from their creation to the present. This review considers the main graphene‐based functional FRP composites producible on a large‐scale and discusses the corresponding manufacturing methodologies for the effective incorporation of graphene materials into them. It also addresses the latest published mechanical and functional results for gFRPs. Subsequently, each proposed material system is benchmarked with some alternative existing technologies, for a specific application area, in the form of a comparison table as well as a radar chart.

We hope this may facilitate bridging the gap between the research knowledge and the technology that has to be developed before laboratory achievements can be successfully translated into pilot plant or industrial‐scale production to achieve higher technology readiness levels (TRLs). Finally, the excellent functional and structural properties of the resulting gFRP composites are highlighted with a view to their future applications.

## 2 Scientific Research and Technological Trends for Graphene Polymer Composites

**Figure 1:**
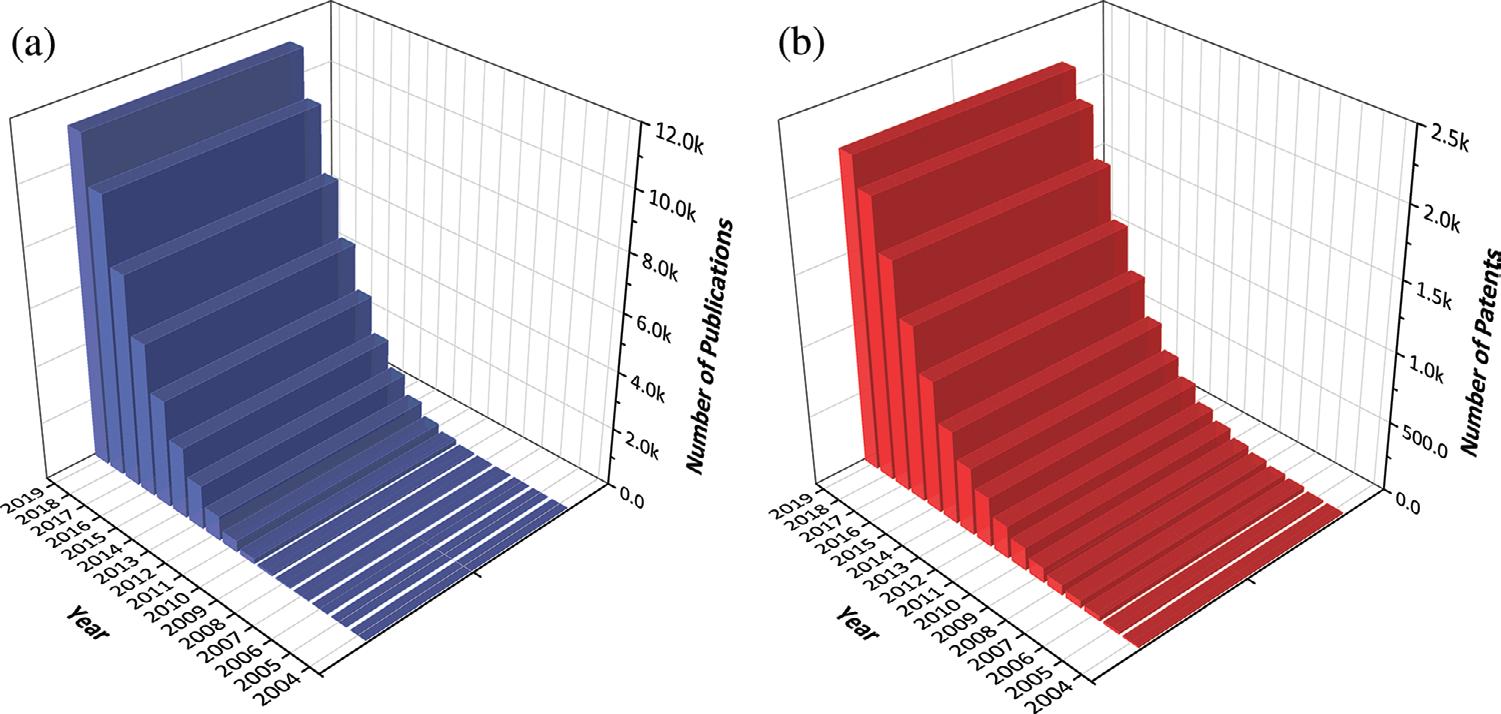
a) The cumulative summary for the approximate number of graphene–polymer composites publications from 2004 to September 2019 (using Web of Science). b) The cumulative summary for patent application “graphene–polymer composites” as a function of application year. Note: patents remain unpublished for up to 18 months from their filing. Accordingly, 2018 and 2019 are under‐represented; Data updated as of June 2018.

To quote the Nobel Laureate Frank Wilczek: “Graphene is probably the only system where ideas from quantum field theory can lead to patentable innovations.”^[^ ^28^ ^]^ Advanced graphene‐enhanced FRP composites possess unique properties that allow for novel and exciting applications in structural fabrication. These are often cost effective, possess high processability in solvents and polymers and have tunable chemical properties.^[^ ^29^ ^]^ They have recently attracted both academic and industrial interest because they can produce a dramatic improvement in properties at very low levels (from 0.5% up to 10% w v^−1^). This is confirmed, in part, by the significant global rise in publication and patenting activities since 2004 by both research centers and universities as well as manufacturers (**Figure** **1**a). The yearly publication rate has increased by an average of over 180% in recent years. This significant growth is, in large part, a testament to the unique set of properties offered by these materials. In addition to these, graphene technology is based squarely on carbon, one of the most abundant materials on earth. It is, thus, an inherently sustainable and economical technology. Consequently, enhancements and developments in the manufacturing technologies give realistic promise of creating new, more powerful and versatile, sustainable, and economically viable materials. Figure 1b indicates that the global intellectual property activity around graphene composites has surged since 2007, mimicking the trend in research in Figure 1a, and strong evidence that research investment worldwide is fueling rapid growth in graphene composite technology.

**Figure 2:**
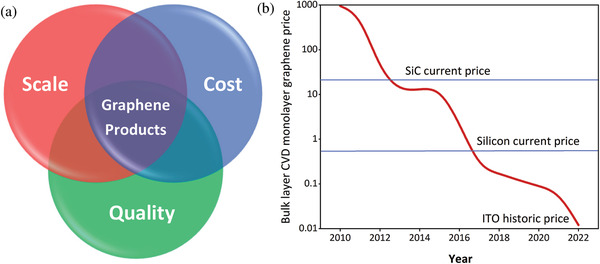
a) Main supply chain factors affecting the rapid uptake of graphene. b) A comparison of the cost of graphene with the benchmark materials since 2010 to 2022. Reproduced with permission.^[^ ^35^ ^]^ Copyright 2020, John Wiley and Sons.

As demonstrated, graphene composite research has emerged as the top research front in materials science in the last decade. However, while graphene has the potential to offer a wide number of solutions to various industrial sectors in the long term, numerous challenges need to be overcome before it can realize its full potential in the marketplace. Supply chain challenges, such as lack of scale up potential, high cost, and the low quality of the generated graphene, have caused the slow uptake of graphene by different industries (**Figure** **2**a).^[^ ^30^ ^]^ In addition, numerous questions should be addressed even in developing a fundamental understanding^[^ ^18^ ^]^ of graphene‐based polymeric composites. Some of the key challenges are listed below^[^ ^31^ ^]^

i) Inconsistencies in key material properties, such as the size, structural integrity and purity of commercial graphene materials^[^ ^32^, ^33^ ^]^ which result in the modification efficiency in composites to fall far below expectations.
ii) Lack of stringent clear standards for graphene characterization, taking into account both the physical properties, safety protocols as well as the specific requirements for a particular application.^[^ ^30^, ^33^ ^]^
iii) Lack of new scalable manufacturing technologies for large‐scale production of graphene‐based customized products.
iv) No existing models of graphene modified composites for high performance structural purposes which cause difficulties in designing graphene composite structures that can offer good mechanical properties as well as predictable and safe failure modes.^[^ ^31^ ^]^
v) Insufficient available data on graphene composites for high‐performance structural applications and interface properties between the graphene and polymer matrix under severe loading conditions.
vi) Little understanding of the potential risks of graphene‐based materials on human health or the environment.^[^ ^34^ ^]^
vii) Lack of specific guidelines and regulations for the disposal of the derived wastes, experimental protocols, and established safety practices.

The state‐of‐the‐art cost‐effective synthesis methods and the emergence of several commercial graphene manufacturers over the past few years have enabled a breakthrough in some of the above‐mentioned limitations. Similar to any other products, the cost versus performance is one of the major concerns for companies when determining whether or not graphene can be used in their products. Since their beginning, the high cost of graphene and the related materials has been considered as one of the major barriers for their entry into the market. Nevertheless, the market price of graphene materials has shown a continual decline over the past decade. Graphenea—a leading graphene supplier—argues that while graphene is currently used for applications that other materials simply cannot support, the price of high‐quality sheet graphene will soon drop below the price of main competing materials.^[^ ^35^ ^]^ Furthermore, the price of graphene is directly linked to its quality, and not all applications require superb material quality. Therefore, recognizing the correct application field for each variant of graphene is of great importance. Besides, the improvements in raw materials and catalysts achieved by big manufacturers, such as Arkema France, Hyperion USA, and Bayer AG, have enabled a noticeable increase in production of carbon‐based nanomaterials including graphene, from the order of grams to tons.^[^ ^36^ ^]^ A comparison of the cost of graphene with the benchmark materials between 2010 and 2022 is given in Figure 2b.^[^ ^37^ ^]^

The Graphene Flagship project is the first of the European Commission's future and emerging technology (FET) which was launched in 2013 whose mission is to address major scientific and technological challenges through long‐term, multidisciplinary research and development efforts. According to their annual report 2018, there are over 350 companies globally producing graphene materials or developing products incorporating graphene and this number is expected to rapidly grow in the coming decade.^[^ ^38^ ^]^ The graphene market has been dominated by North America, accounting for a large share in the global market, followed by Europe. However, as stated by PRNewswire, Asia‐Pacific is anticipated to be the fastest growing region for adoption of graphene products. According to the latest report “Global Market for Graphene 2018–2027” released by PR Newswire, the global graphene composite market is expected to grow from USD 50.24 million in 2018 to USD 782.11 million by 2026 at a compound annual growth rate (CAGR) of 24.67% during the forecast period from 2019 to 2026.^[^ ^39^ ^]^ Factors propelling the growth of the market include increasing demand for graphene nanocomposites, remarkable characteristic properties, and helpfulness in simplifying the next generation of technologies in various industries. Capacitor‐based technologies are also expected to grow from USD 50.0 million in 2020 to USD 625 million by 2025, a CAGR of 65.7%. Structural materials, as a market sector, should reach USD 68.4 million by 2020 and USD 340 million by 2025, registering a CAGR of 37.8% for the period.^[^ ^40^ ^]^

**Figure 3:**
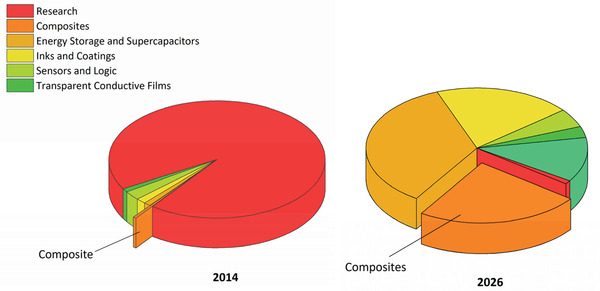
Market snapshot in 2014 and 2026 by IDTechEx. This will change as real applications sales grow. Source: www.IDTechEx.com/graphene.^[^ ^43^ ^]^

The market will be segmented across many applications, reflecting the diverse properties of graphene. Functional inks and coatings made up about 21% of the market in 2018. It is predicted that energy storage and composites will grow to be the largest sectors, ultimately, controlling 25% and 40% of the market in 2027, respectively.^[^ ^41^ ^]^ This is mainly due to the higher demand for fuel efficient vehicles and stricter government regulations. Additionally, environmental regulations to lower CO_2_ emissions in the Europe and the America will continue driving the adoption for graphene‐based materials in the coming years. IDTechEx has also reported a graphene market value of roughly USD 200 million by 2026, where more than 75% of the market share will belong to composites, energy storage (supercapacitors), and inks and coatings, whereas sensors, logic, transparent conductive films, and research applications will altogether take up less than 15% of the market share (**Figure** **3**).^[^ ^42^ ^]^

**Figure 4:**
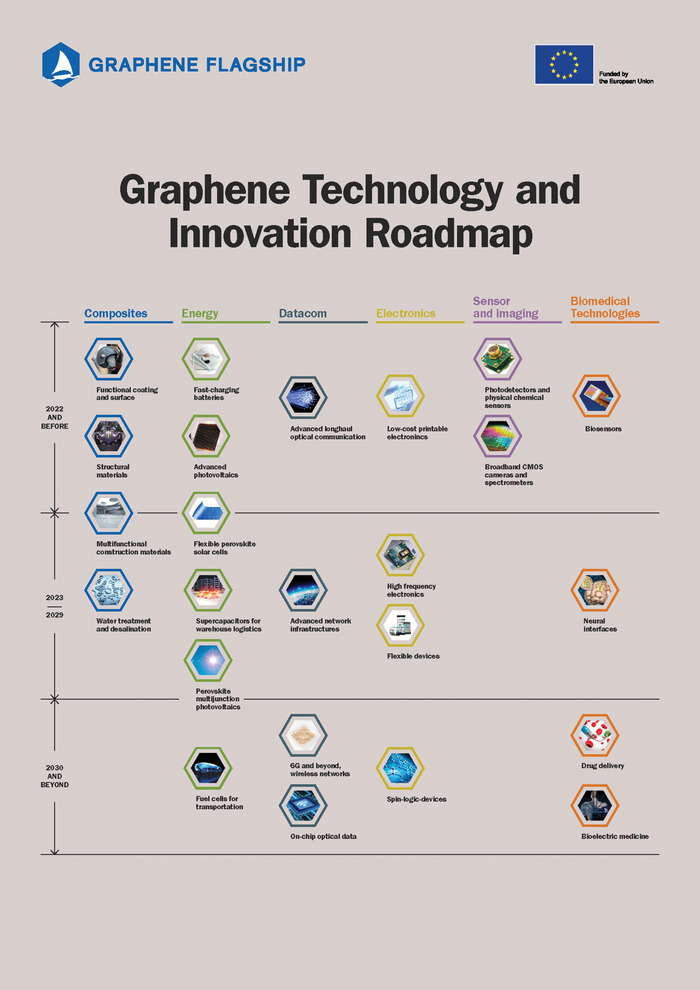
Graphene applications roadmap. Adapted with permission.^[^ ^38^ ^]^ Copyright 2019, Graphene Flagship.

Graphene commercialization is still very much in its infancy. Generally speaking, applications that require high‐quality graphene are expected to take longer to reach the market, as evidenced by recent application predictions (see **Figure** **4**).^[^ ^38^ ^]^ Applications that demand fast electronic performance, which were first thought to be the most promising applications for graphene, are, surprisingly, anticipated to grow very little in the short term, mostly due to the fierce competition that graphene faces from established materials such as silicon and indium‐tin‐oxide. Recent product launches include graphene‐based bicycle and automotive tires, smart phone batteries, supercapacitors, water filtration membranes, and composites. Major corporations including Ford and Huawei have recently integrated graphene into their products and there have also been several product launches in the areas of batteries, supercapacitors, textiles, and sportswear.^[^ ^41^ ^]^

## 3 Scalable Production Strategies of Graphene Materials

The improved properties offered by graphene polymer composites have driven up the demand for their use in a variety of structural and industrial applications. Increasing TRL, material quality, consistency, and cost have remained the key pillars for advancement toward commercialization. The large scale, single layer synthesis of graphene was attempted as early as 1975^[^ ^44^ ^]^ when Lang showed formation of mono‐ and multilayered graphite by thermal decomposition of carbon on single crystal Pt substrates. Most current graphene production methods are not yet scalable to the large quantities required to guarantee their long‐term supply. The bulk production methods that do exist have inherent limitations concerning the quality of graphene produced, which limits its potential range of applications. Furthermore, even though the production costs for graphene have fallen considerably over the past few years, it is still expensive compared to other material costs.

One advantage of graphene over other nanomaterials is that it can be produced either by bottom‐up assembly of smaller atoms and molecules (construction) or top‐down exfoliation of graphite stacks (destruction).^[^ ^45^ ^]^ It is widely known that graphene quality and its intrinsic properties are strongly affected by the synthesis method chosen.^[^ ^46^ ^]^ Therefore, a number of physical, chemical and electrochemical approaches have been tested, including micromechanical cleavage, chemical exfoliation and chemical vapor deposition (CVD).

**Figure 5:**
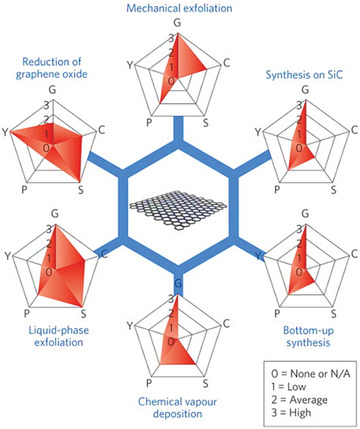
Some common production methods of graphene in relation to quality and scalability potential. Each method has been evaluated in terms of graphene quality (G), cost aspect (C; a low value corresponds to high cost of production), scalability (S), purity (P), and yield (Y) of the overall production process. Adapted with permission.^[^ ^45^ ^]^ Copyright 2020, Springer Nature.

Top‐down methods are generally highly scalable and produce high quality products, but have difficulties in forming products with consistent properties, low yield and rely heavily on the finite graphite precursor. For instance, micromechanical cleavage is considered as one of the most effective and reliable methods to produce high‐quality graphene. Many of graphene's remarkable properties including high electron mobility at room temperature (200 000 cm^2^ V^−1^ s^−1^),^[^ ^47^ ^]^ exceptional thermal conductivity (5300 W m^−1^ K^−1^),^[^ ^46^ ^]^ and superior Young's modulus (YM) (1 TPa) and ultimate tensile strength (UTS) (130 GPa)^[^ ^48^ ^]^ have been verified using this method.^[^ ^8^ ^]^ However, the poor solution‐processability of pristine graphene, the low yield rate of this method and limited scalability hinders its practical application in the large‐scale manufacture of industrial composites. On the other hand, even though the bottom‐up methods produce graphene products with almost defect‐free and large surface area, they often involve high production cost and sophisticated operational setup. At present, the main preparation methods of graphene include mechanical exfoliation, liquid phase stripping, oxidation‐reduction method, and chemical vapor deposition.^[^ ^49^ ^]^ **Figure** **5** summarizes the key characteristics of the most common graphene production methods.^[^ ^45^ ^]^

**Figure 6:**
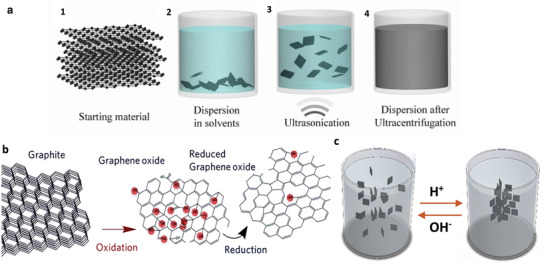
a) Liquid phase exfoliation of graphite. 1) Starting material (graphite), 2) chemical wet dispersion, 3) ultrasonication, and 4) final dispersion after the ultracentrifugation process. Reproduced with Permission.^[^ ^51^ ^]^ Copyright 2019, OSA Publishing. b) Schematic showing the reduction process of GO. Adapted with Permission.^[^ ^52^ ^]^ Copyright 2019, IOP Publishing. c) the solution–gelation transition of graphene sheets upon switching pH values.

Currently, two methodologies are broadly employed for the bulk production of graphene: liquid‐phase exfoliation and reduction of graphene oxide (GO). In liquid‐phase exfoliation, pristine or expanded graphite particles are first dispersed in a solvent to reduce the strength of the van der Waals attraction between the graphene layers. An external driving force such as ultrasonication, electric field or shearing is then used to induce the exfoliation of graphite into graphene sheets (see **Figure** **6**a). In the second method, GO, is produced by strong oxidation of pristine graphite^[^ ^50^ ^]^ followed by stirring or ultrasonication in liquid media. GO is then subsequently reduced to graphene to restore its π network (see Figure 6b).^[^ ^46^ ^]^ Chemical, thermal and electrochemical processes are commonly employed in this order to produce reduced graphene oxide (rGO).

GO is heavily oxygenated compared to pristine graphite and so is highly hydrophilic and readily exfoliated in water, yielding stable GO dispersions consisting mostly of single layered sheets. After chemical or thermal reduction, the flat graphene sheets tend to restack and agglomerate together (see Figure 6c). This phenomenon negatively affects their surface area to volume ratio and, consequently, limits their effectiveness as fillers for composites. Despite the low‐to‐medium quality of the obtained material due to the presence of both intrinsic defects (edges and deformations) and extrinsic defects (O‐ and H‐containing groups), solution processing methods provide an environmentally friendly, low‐cost and scalable approach to produce graphene sheets in bulk quantities.

Recent research has also indicated that nanocellulose can act as an excellent aqueous dispersion agent for the dispersion of graphene in water.^[^ ^53^, ^54^ ^]^ One other limitation is the lack of practical knowledge on the optimum aspects of the processing equipment, such as the extruder design (e.g., location of high‐pressure pump and vacuum valve, screw element design, etc.) and control of water (or solvent) flow and volume which limits the use of the solution processing technique for the production of high quality graphene–polymer composites.

The use of graphite nanoplatelets (GnPs) in powder form has recently become one of the most effective, low‐cost, and scalable approaches for the production of graphene/polymer composites.^[^ ^55^, ^56^, ^57^, ^58^, ^59^ ^]^ XGSciences and Cheap Tubes are two commercial graphene suppliers able to provide inexpensive powder solutions at an industrial‐scale. Dry GnPs are commonly used as additional nanofillers into the polymer matrix to induce multiple functional properties. Chemical compatibility between the filler and the matrix is a critical factor to consider in the preparation of a composite material.^[^ ^60^ ^]^ The ultrahigh surface area of GnPs allows a large surface contact area with the polymer, resulting in enhancement of the composite functional properties. However, as discussed earlier, the large surface area of the GnPs planar sheets also encourages agglomeration. Consequently, this process usually requires the application of high shearing forces in order to achieve a uniform dispersion of GnPs within the polymer matrix. A detailed understanding of rheological and dispersion properties is also essential to obtain optimal dispersion conditions. It has been reported that while entanglement of CNTs in a polymeric matrix at higher loadings results in undesirable viscosity increases, GnPs can more easily slide past one another moderating the viscosity increase, even at relatively higher proportions, and thus become more favored than CNTs from a processing point of view in the production of polymer composites.^[^ ^60^, ^61^ ^]^ Some graphene production methods are depicted in Figure 5, and each method can be utilized depending on the application and desired quality of graphene.

## 4 Manufacturing Strategies for gFRPs

**Figure 7:**
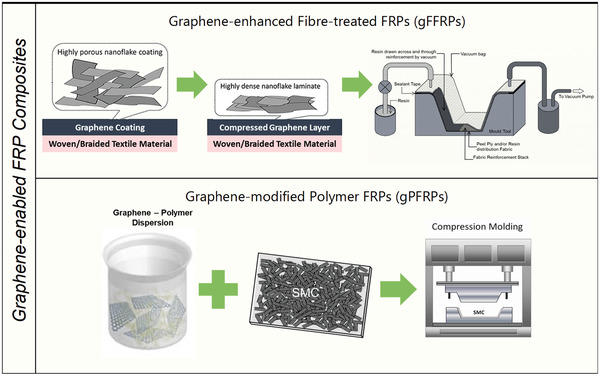
Schematics of the two proposed strategies for multiscale composite manufacturing.

The integration of novel graphene materials will benefit the development of advanced FRP composites with improved structural and functional properties that allow for novel and exciting structural applications. Based on a survey of the available literature, gFRPs may be categorized into three main sub‐groups based on their engagement type within the composite. The first category may be referred to as “Graphene‐modified Polymer FRPs” (gPFRPs), also known as nanoaugmented FRPs, which are fiber‐reinforced composites wherein graphene nanoparticles are randomly dispersed into the polymer matrix of the composite material. To the best of the authors' knowledge, gPFRPs have occupied the vast majority of studies in the field of gFRPCs. The second group encompasses reinforced composite fabrication wherein graphene is integrated into FRPs either by being doped on fiber reinforcement or inserted as large‐area layers in the form of a coating, based on the final modification purposes. This group is referred to as “graphene‐enhanced fiber‐treated FRPs” (gFFRPs), in other words, nanoengineered^[^ ^7^ ^]^ FRPs. The third category involves a combination of the two above methods to introduce graphene nanomaterials onto a fiber surface as well as within the polymer matrix to boost the material's performance. gPFRPs and gFFRPs are discussed in more detail in the following sections. (**Figure** **7**)

A number of manufacturing approaches have been employed to create multiscale gFRPs with tunable properties. It has already been noted that the manufacturing process of composites has a critical effect on their final properties. Whether graphene is embedded in the matrix or on the fiber (or both), the selection of the manufacturing method for a particular application is largely determined by the polymer properties, as well as the fiber size and geometry. Reinforcing fibers may be introduced either in the form of discontinuous or continuous fibers (random or oriented) or in the form of a mat. Integration of short fibers into composites would enable the creation of advanced nanocomposite matrix materials to improve the bulk properties of FRPs and introduce new functionalities, while embedding continuous fibers would facilitate the manufacture of scalable laminate‐structured composites. The polymer matrix to be used may be either a thermoset or thermoplastic polymer (or a combination of both) depending on the ultimate end‐use. Thermoplastics are cooled to a temperature at which they solidify, while thermosetting polymers are cured by crosslinking the molecular chains in order to create a solid structure.^[^ ^62^ ^]^

gPFRPs can be made through standard closed‐molding techniques (compression molding or reinforced reaction injection molding (RRIM)) or novel 3D‐printing technologies. Compression molding is the most frequently used technique for making gPFRP parts with random fiber placements, whereas RRIM or 3D printing are employed in cases where the fibers are (usually) randomly oriented short fibers dispersed in a polymeric matrix. Laminate‐structured gFFRPs, on the other hand, can be manufactured using both closed and open‐molding technologies. Hand lay‐up is the most common and least expensive of the open‐molding methods because it requires the least amount of equipment, while filament winding is an automated process that makes high strength‐to‐weight ratio laminates and provides a high degree of control over uniformity and fiber orientation. Vacuum bagging (VB), vacuum‐assisted infusion processing (VIP), and resin transfer molding (RTM) are the known closed‐molding routes which can be also applied for the manufacturing of gFFRPs. In general, while the inclusion of graphene materials into FRPs has shown promising results that significantly improve their properties, the need for additional processing steps may be disadvantageous. Addition of graphene materials into a polymer matrix can create complexities concerning dispersion viscosity control, dispersion flowability within the mold and changes in the curing rate. Integration of graphene materials onto the fiber component can introduce further variables such as uneven coating throughout the laminate, improper bonding between the layers and penetration of resin into the fibers. It is worth mentioning that the manufacturing strategy selected for gFRPs is also strongly influenced by the industrial sector for which they are bound and the rate of production required.^[^ ^63^ ^]^

### 4.1 gPFRPs

Development of gPFRPs is a promising strategy that is attracting a great deal of interest for possible use in a broad range of high end applications (including the aerospace and automotive industries)—all due to the unique blend of properties they offer for the development of intricate hybrid and multipart designs.^[^ ^64^, ^65^ ^]^ gPFRPs can be used either solely as nanocomposites (only with nanoscale fiber reinforcement and/or other nanoparticles) or employed as an advanced matrix with improved bulk properties to introduce new functionalities in composites having reinforcement at other scales, such as long fiber/fabric reinforced polymer composites.

In this method, graphene, in the form of platelets or dispersions, is embedded into a polymer matrix. Traditional macroscopic fibers are then embedded within the graphene‐based composite, either in the form of short fibers integrated into the polymer matrix to create nanocomposites or as continuous fabrics to develop laminate‐structured composites.^[^ ^29^ ^]^ The chemical and mechanical properties of FRP composites strongly depend on the composition of the constituent fiber as well as fiber sizing. The fibers that may be used include, but are not limited to GFs, CFs, and basalt fibers (BFs).^[^ ^66^, ^67^, ^68^, ^69^, ^70^, ^71^, ^72^, ^73^ ^]^ GFs are the most widely used reinforcement among all the synthetic fibers which can be found in the form of roving's, chopped strand, yarns, fabrics, and mats.^[^ ^74^ ^]^ GFs offer excellent strength and durability, thermal stability together with resistance to impact, chemical, friction, and wear properties. However, the machining of glass fiber‐reinforced polymers (GFRPs) is relatively slow, challenging, and shows reduced tool life while working on conventional machining systems.^[^ ^74^ ^]^ GFs also carry the disadvantage of disposal at the end of their service life.^[^ ^75^ ^]^ GFs are used in the manufacture of structural composites, printed circuit boards and a wide range of special‐purpose products.

CFs are employed instead of GFs in applications where more stiffness is required. CFs have several advantages including high stiffness and tensile strength (TS), lightweight, high chemical resistance, excellent temperature tolerance and low thermal expansion.^[^ ^76^ ^]^ Carbon is also known to have better resistance against chemical attack than BFs and GFs. Both basalt and glass fiber have silica as their major constituent, and the chemical‐degradation mechanisms are dominated by silica's susceptibility to chemical attacks.^[^ ^77^ ^]^ These properties have made CFs quite popular in aerospace, automobile, civil engineering, military, and sports applications. However, they are relatively pricy when compared with either GFs or BFs. Besides, due to their inherent brittleness, the key disadvantage associated with the use of carbon fibers is the catastrophic failure of the resulting composites.^[^ ^78^ ^]^ During the last years, BFs came into the focus of researchers as a cost‐competitive fiber with excellent temperature resistance and slightly higher mechanical properties, and thermal and chemical stability compared to those of E‐glass fibers.^[^ ^79^ ^]^ Unlike GFs, BFs do not split into finely disperse (less than 0.4 µm) microfiber structures with carcinogenic properties under high loading conditions. However, as a natural product, the chemical composition and thus physiochemical properties of BFs vary significantly with the mining region.^[^ ^79^ ^]^ Furthermore, BFs have indicated extremely low thermal conductivity which will add several complexities in composite manufacturing.^[^ ^78^ ^]^ **Table** **1** compares the major physiomechanical characteristics of as‐discussed fiber types.

**Table 1:** Basic physiomechanical properties of GFs, CFs, and BFs^[^ ^78^ ^]^

Fibers	Density, ρ [kg m^−3^]	Diameter [µm]	Tensile strength, σ_t_ [MPa]	Young's modulus, *E* [GPa]
Glass	2500–2600	10–15	1500–2700	80–90
Carbon	1780	8	2800–5000	230
Basalt	2800–3000	9–17	2600–3200	93–110

**Figure 8:**
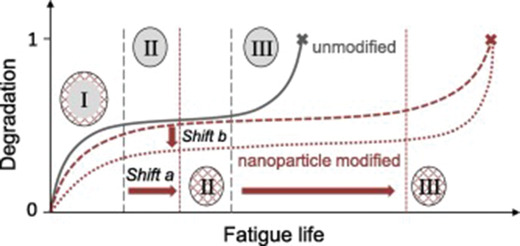
Schematic of desired changes in degradation development with typical three degradation phases for FRP (unmodified) and nanoparticle modified FRP loaded in fatigue: fatigue life improvement by an extension of phase I and II (shift a) and a less pronounced degradation increase (shift b). Adapted with permission.^[^ ^84^ ^]^ Copyright 2019, Elsevier.

The combination of the micro–nano filler types may prove advantageous for the final product since the ultimate properties can be a result of additive or synergistic effects between the fillers. Moreover, the hybridization procedure can counterbalance some of the disadvantages of a specific reinforcing filler, while it may also enhance the interactions with the matrix depending on the functionalization route.^[^ ^80^ ^]^ This multifunctionality is a very important consideration in the preparation of hybrid composites since the combined properties of the micro–nano fillers can lead to the production of a new material with quite a unique set of properties. Within these novel materials the stress can be transferred from the microscale to the nanoscale reinforcement, improving the ultimate mechanical and fatigue properties.^[^ ^81^ ^]^ The fatigue lifetime can be significantly increased by the addition of small amounts of graphene nanoparticles.^[^ ^82^, ^83^ ^]^ In accordance with this, the fatigue degradation process was found to progress more slowly and at a lower scale.^[^ ^84^ ^]^ This effect may be due to an increase in energy absorption and a shift in the dominant damage mechanism for modified FRPs depending on the level of fatigue load (see **Figure** **8**a). Phase I is characterized by initiation and growth of matrix cracks in between the reinforcing fibers in off‐axis layers, which leads to a rapid increase of FRP degradation. In phase II a saturation of transverse matrix cracks is reached and delamination and longitudinal inter fiber fracture begin to form. The degradation rises almost constantly but slowly, before increasing rapidly again in phase III until global failure of the composite occurs due to delamination growth and fiber fractures.^[^ ^85^, ^86^ ^]^ It is evident that the whole degradation process starts out with damage in the fiber embedding polymer matrix and, thus, matrix modification is considered a promising approach to improving the fatigue performance of reinforced polymer composites. Furthermore, the ultimate cost of the final product may be reduced since well‐established microscale reinforcements such as CFs^[^ ^69^, ^70^, ^87^, ^88^ ^]^ and GFs^[^ ^81^ ^]^ can be combined with small amounts of graphene nanomaterials to produce hybrid composites.

Both thermosetting and thermoplastic polymers can be used for the matrix material. Common thermoset polymer matrix materials include polyester (PES), vinyl ester and epoxy whereas poly ether ether ketone (PEEK), polyurethane (PU), polyethylenimine (PEI), and polyphenylene sulfide (PPS) are thermoplastics frequently used in the composite manufacturing industry.^[^ ^89^ ^]^ Thermoplastic and thermoset materials are both processed at elevated temperatures. Thermoplastics are normally in a rigid or hardened state and soften as temperature is increased toward a material's melting point. Many thermoplastic polymers have shown an increased impact resistance (as high as ten times) over comparable thermoset composites.^[^ ^89^ ^]^ The other major advantage of thermoplastic composites is their ability to be reformed and reshaped when heated, which is not possible with thermoset resins. This also allows for the recycling of the thermoplastic composite at the end of their functional life. In addition, thermoplastic composites can be fabricated by techniques which are less cumbersome and potentially faster than current curing systems in an autoclave. However, due to the initial natural solid state of thermoplastic resins, it is much more difficult to impregnate the reinforcing fiber before applying heat and pressure.^[^ ^90^ ^]^ Thus, the process of making the composite typically requires special tooling and equipment and is, therefore, more complex and expensive. Thermoset resins begin in a liquid state and harden as a result of the completion of a thermochemical reaction. The permanent crosslinks created make the cured material stronger, more dimensionally stable, more highly resistant to heat and chemicals and with a higher degree of rigidity than other materials. On the downside, many thermosets have limited shelf lives prior to the cure compared to thermoplastic materials, which are stable at room temperature. Every thermoset mold may be considered as a chemical reactor—each part cycle is a polymerization—and, thus, must be carefully controlled to avoid overheating and to achieve the desired state of cure.^[^ ^91^, ^92^ ^]^

More recently, ductile thermoplastics are being investigated as tougheners for brittle resins to enhance their impact resistance and toughness performance.^[^ ^91^, ^92^ ^]^ This route could be regarded as a way of fast‐tracking the incorporation of graphene‐based materials into a range of high end applications such as the aerospace and automotive industries.^[^ ^42^, ^93^ ^]^ However, a crucial step is the dispersion of the carbon nanofillers. A well‐dispersed state ensures a maximized reinforced surface area, which will affect the neighboring polymer chains and, consequently, the properties of the whole composite. Moreover, the bonding between the individual fillers plays a very important role on the final physicochemical properties of gFRPs.

#### 4.1.1 Processing Routes for Preparation of Polymer–Graphene Dispersions

A lot of effort has been put into the production of well‐dispersed hybrid material systems using either graphene‐based suspensions or dry graphene materials enabling multifunctional graphene‐based polymer composite fabrication.^[^ ^94^, ^95^, ^96^, ^97^, ^98^, ^99^ ^]^ Due to the low cost and high yield production of GO and rGO, a majority of graphene/polymer material systems investigated are fabricated using either GO or rGO (thermally or chemically) as fillers. However, similar to the inclusion of other nanofillers like CNTs and regardless of the polymer matrix used, the major challenge lies in the proper processing of graphene composites in order to achieve a uniform dispersion of GnPs within the polymer matrix.^[^ ^100^ ^]^ Three main approaches have been employed and developed as scalable and economic approaches to preparing well‐dispersed graphene polymer matrices—these are chemical or mechanical methods or a combination of both. Solution mixing and melt blending are considered as mechanical dispersion tools where the polymer matrix and the filler interact through relatively weak dispersive forces, while in situ polymerization is a chemical approach which enables linkages between the graphene‐based filler and the supporting polymer to promote their interfacial bonding.^[^ ^46^ ^]^ These methods are briefly discussed below.

##### Mechanical Dispersion Methods

Solution mixing is the most commonly used method for large‐scale preparation of graphene‐based polymer dispersions and may be applicable for thermoset and thermoplastic polymers.^[^ ^101^ ^]^ Solution‐based methods generally involve the mixing of graphene nanoparticles, colloidal suspensions of GO platelets or other graphene‐based materials with the desired polymer, either itself already in solution or by dissolving the polymer in the suspension of GO platelets, through sonication, simple stirring or high‐shear mixing. The dispersion method is particularly important as graphene sheets are prone to crumple, wrinkle, and roll during processing.^[^ ^102^ ^]^ GO contains carboxylic, hydroxyl, and epoxy groups on its surface which can significantly alter the interactions between the layers of graphene and improve their dispersion in water and, thus, GO sheets can be easily dispersed in aqueous media and composites with hydrophilic characteristics.^[^ ^46^ ^]^ However, it is well known that oxygen‐containing functional groups attached to the GO surface render it electrically insulating, limiting its applicability for conductive polymer‐based composites. The electrical conductivity can be significantly increased through chemical or thermal reduction, however irreversible restacking of graphene sheets after the reduction process can significantly reduce their effectiveness. Consequently, the uniform dispersion of individual graphene sheets within a polymeric matrix becomes crucial in the fabrication of good quality graphene polymer dispersions.^[^ ^46^ ^]^ Poly(vinyl alcohol),^[^ ^103^ ^]^ polystyrene,^[^ ^94^ ^]^ poly(methyl methacrylate) (PMMA),^[^ ^95^ ^]^ Nafion,^[^ ^104^ ^]^ and PU^[^ ^96^ ^]^ are just some of the polymers that have been explored to date for their suitability in the production of graphene composite materials. An additional benefit of solution mixing is that the viscosity of the graphene–polymer dispersion is lower than those prepared by other blending techniques, and this allows the polymer chains to intercalate more easily between the graphite layers during the preparation process. On the downside, in the case of using an already‐dispersed suspension of graphene for preparing graphene–polymer dispersion, there may be difficulties in removing solvent traces and trapped air bubbles for achieving high‐performance properties.

Melt blending is another versatile method used to prepare graphene‐based polymer dispersions using thermoplastic polymers. This approach is believed to be more environmentally friendly (since it is solvent‐free), economical and suitable for mass production of graphene polymer composites. In this process graphene is typically blended with melted polymers in a twin‐screw extruder to produce hybrid material dispersions as a result of the polymer chains becoming intercalated or exfoliated.^[^ ^97^ ^]^ Compared with other commercially available reinforcing fillers, including CFs, carbon black and clays, the smaller aspect ratio of the GnPs provides a better compatibility with the polymer matrix and favors the formation of more uniform composites.^[^ ^8^ ^]^ Drawbacks of this approach are additional challenges associated with separating nanoparticles in a viscous medium and achieving a homogenous dispersion. Composite materials based on PU, polyethylene terephthalate (PET), polylactic acid (PLA) and polycarbonate (PC)^[^ ^98^ ^]^ have been developed by melt blending with GO‐based dispersions.^[^ ^98^ ^]^

##### Chemical Dispersion Method

**Figure 9:**
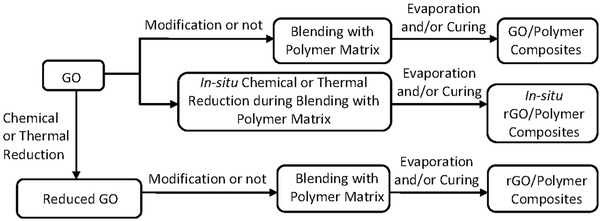
The general fabrication routes for graphene‐based polymer dispersions with GO or rGO. Reproduced with permission.^[^ ^46^ ^]^ Copyright 2019, John Wiley and Sons.

In situ polymerization is also a very efficient method for uniformly dispersing rGO within the polymer matrix while also creating strong interactive links between these two components. This method generally involves mixing of a filler in the neat monomer (or multiple monomers), or a solution of monomer, followed by polymerization in the presence of the dispersed filler. These steps are often followed by precipitation/extraction or solution casting to generate composite samples. Using this approach, composites with either covalent or noncovalent linkages between the matrix and filler are achievable. In situ polymerization has been achieved with a variety of polymers, such as epoxy,^[^ ^105^, ^106^ ^]^ PU,^[^ ^107^ ^]^ polyamide,^[^ ^108^ ^]^ poly(ethylene) (PE),^[^ ^109^ ^]^ PMMA,^[^ ^110^ ^]^ and Poly(pyrrole) (PPy).^[^ ^111^ ^]^ Using this approach, a high level of dispersion of the graphene‐based filler has been achieved without a prior exfoliation step. A comparison has been conducted between the effect of different dispersion preparation methods (solution mixing, in situ polymerization and melt blending) on the conductivity of graphene‐enabled nanocomposites.^[^ ^112^, ^113^ ^]^ Kim et al. investigated how different processing methods affect the conductivity of PU/graphene nanocomposites. They found that the highest conductivity values were obtained from composites prepared through solution blending. Melt mixing lead to particle re‐aggregation and attrition which will reduce the lateral size of the graphene. In situ polymerization caused covalent bonds to form between the matrix and the filler, which hindered direct contact between the fillers and reduced the effective aspect ratio. The general dispersion routes for the preparation of graphene polymer dispersions using GO or rGO as fillers are summarized in **Figure** **9**.

## 5 Current Achievements in the Fabrication of gPFRPs

Interest in the field of gPFRPs has been mainly driven by the high demand for materials that simultaneously perform structural and nonstructural functions capable of satisfying specific, and often multifaceted, performance requirements. For instance, fiber‐dominated properties (i.e., longitudinal TS and elastic modulus) of conventional unidirectional composites with micron size fiber reinforcements are excellent, whereas the corresponding matrix‐dominated transverse TS and longitudinal compressive strength properties are typically poor. Replacing the neat polymer matrix with a modified nanocomposite matrix has been one of the key strategies to significantly improving such unfavorable properties. To this end, either graphene or functionalized graphene are incorporated into resin to amend the matrix properties.

Since the first report of the preparation of a polystyrene–graphene composite in 2006,^[^ ^114^ ^]^ several property enhancements were reported as a result of the inclusion of graphene materials into either thermosetting or thermoplastic polymer matrices. To that end, some thermosetting polymers such as Bismaleimide (BMI),^[^ ^115^ ^]^ PES,^[^ ^116^ ^]^ etc. have been investigated; however, in the past decade or so, the major scientific activities for preparing gPFRPs were focused on epoxy. Epoxy‐based graphene composites were first reported in 2007^[^ ^61^ ^]^ where Yu et al. employed a multistep approach including acid intercalation, thermal exfoliation, physical separation and dispersion to convert natural graphite into few‐layer GnPs. A high‐shear mixing approach was then used to come up with a homogeneously dispersed mixture of epoxy and GnPs. As‐fabricated composites demonstrated enhanced thermal conductivities (with values up to 1.45 W mK^−1^ at low‐volume GnPs loadings (≈5 vol%)) which significantly outperformed CNT‐embedded composites.

Since then, extensive advances have been made in the fundamental understanding of graphene‐incorporated composites. Although only gPFRPs are considered in this review, it is worth noting that several other polymer–graphene nanocomposites have been reported.^[^ ^106^, ^117^, ^118^, ^119^, ^120^ ^]^ Researchers have attempted to prepare gPFRPs adding various concentrations of graphene materials from 0.05 to 15 wt% within the epoxy, mostly through the aid of solution mixing approaches including, but not limited to, mechanical mixing, ultrasonication, a three‐roll‐mill process or a combination of those, to achieve enhanced matrix properties. Based on the available literature, a substantial number of studies carried out on gFRPs were aimed at providing enhanced structural characteristics compared to those prepared using an unmodified resin. In addition to this, graphene fillers have been integrated into the resin either in the form of a dry GnPs powder or dispersion. Shen et al. were probably the first to consider the fabrication of graphene‐enabled carbon fiber‐reinforced polymer (CFRP) composites through the direct addition of GnPs into the matrix material followed by a solvent type prepreg process and analyzed the role of graphene on the impregnation of fibers and prepreg processing conditions.^[^ ^121^ ^]^ In this study, various amounts of GnPs (0.25–1.5 wt%) were uniformly dispersed in epoxy resin via a mechanical mixing method. Significant improvements in the mechanical properties (UTS, flexure, and fatigue life) were attained for these epoxy resin composites and CF‐reinforced epoxy composite laminates. The fatigue life of epoxy/CF composite laminate with GnPs‐added 0.25 wt% was increased over that of neat laminates at all levels of cyclic stress. This was likely due to the formation of a significantly improved adhesion between the fibers and resin as a result of addition of GnPs, which suppressed the formation of microcracks during cyclic loading. In addition, an increased TS, flexural modulus (FM), and flexural strength (FS) were also achieved and this approach was subsequently applied by a variety of researchers to achieve modified composite properties. Moriche et al. have analyzed the electrical behavior and mechanical properties of –NH_2_ functionalized GnPs‐reinforced epoxy composites of the graphene filler once scaled up from a nanocomposite to a multiscale composite material. The electrical conductivity of the reinforced nanocomposites was reported to be in the order of 10^−4^ S m^−1^, in contrast to the in‐plane conductivity of multiscale composite materials with the same GnPs content, which was found to be ≈10^−3^ S m^−1^.^[^ ^122^ ^]^ Kandare et al. have lately evaluated both functional and mechanical properties of CFRPs through the simultaneous inclusion of GnPs and silver nanoparticles/nanowires and reported 40% enhancement in the through‐thickness thermal conductivity at a combined loading of 1 vol%, while the inclusion of GnPs alone at the same loading resulted in only 9% improvement. Similarly, the through‐thickness volume resistivity of CF/epoxy laminates incorporating GnPs together with silver nanoparticles/nanowires was notably reported to be lower (≈70%) than can be achieved by GnPs alone (≈55%). Small improvements were found, though, in the mechanical properties, with CF‐reinforced laminates exhibiting 51.0 GPa YM, 639 MPa TS, 68.0 GPa FM, 679 MPa FS, 11.9 GPa compression modulus (CM), and 387 MPa compression strength (CS).^[^ ^123^ ^]^

In 2012, Ma et al. were the first to demonstrate the great potential of GO‐modified epoxy as a functional coating for GFs. Their results show that when graphene is used as a nanoscale filler in the epoxy, the nanocomposite coating functions as a barrier layer to prevent GFs from environmental attacks.^[^ ^124^ ^]^ Shortly after, Mannov et al. looked into the improvement of the matrix toughness via the integration of thermally reduced GO (TrGO) for use within the laminate‐structure composites. In this study, 0.3 and 0.5 wt% of TrGO were dispersed in an epoxy using a three‐roll mill. CFRP and GFRP prepregs based on such modified epoxy were then manufactured using a filament winding technology, followed by the manufacture of laminates in an autoclave. The residual compressive properties (from compression after impact (CAI) test) of the as‐processed laminates were reported to be improved significantly. The CFRP specimens with 0.3 wt% TrGO matrix modification showed a 19% increase in residual compressive strength whereas the GFRP composites showed a higher improvement (up to 55%) with 0.3 wt% TrGO dispersed in the resin.^[^ ^125^ ^]^ Recently, Rafiee et al. described a novel approach based on vacuum assisted resin transfer molding (VARTM) to incorporate GnPs, GO, rGO, and MWCNT into the composite laminates for the enhancement of their thermal properties. The results showed that, compared with the neat epoxy/fiberglass composite, improvement in thermal conductivity of fiberglass/epoxy modified with MWCNTs 0.3%, GnPs 1%, GO 2%, and rGO 0.042% were 8.8%, 12.6%, 8.2%, and 4.1%, respectively. It was also concluded that, for the same volume fraction of nanoparticles, the thermal conductivity improvement in GnPs‐modified composites is more pronounced compared with other nanoparticles.^[^ ^56^ ^]^

Few reports exist that consider the use of a graphene‐modified thermoplastic as the matrix for fiber‐reinforced composites (in comparison to those on thermoset gPFRP laminate composites),^[^ ^93^, ^126^, ^127^ ^]^ particularly where continuous fibers or fiber fabrics are used. It can be relatively easier to process thermoplastics based gPFRPs compared to their thermoset counterparts in terms of their manufacturing process. Additional advantages are that there is no need for adding hardener and curing is carried out at ambient temperature. However, the high viscosity of thermoplastic melts and the rapid solidification below the melting point are key factors which would hinder full fiber impregnation. To overcome this issue, either in situ polymerization or solution mixing methodologies are employed to prepare graphene‐modified thermoplastics. One study describes the use of a modified thermoplastic polymer in which the effect of the addition of graphite additive into polyamide 12 (PA12) on both electrical conductivity and fracture toughness behavior of CF/epoxy laminate composites is investigated.^[^ ^127^ ^]^ The focus of this study was on the evaluation of various PA12 inserts (with added graphite) within the composite and the consequent effects on the electrical conductivity and fracture toughness behavior of the final composites. The results showed that the conductivity improves with increasing graphite content; 20 wt% graphite content gives 250% higher conductivity than unmodified PA12, and 30 wt% delivers a 500% improvement (up to >10 S m^−1^). On the other hand, it revealed that the high concentration of the additive also negatively affects the fracture toughness. In addition, it turns out that the relatively high electrical conductivity of the modified PA12 matrix does not carry over into an equivalent improvement in the electrical conductivity of the composites. Furthermore, no enhancements were reported regarding the fracture toughness of CFRP composites through the addition of the graphite to PA12. **Table** **2** summarizes investigations into both thermoset and thermoplastic gPFRPs.

**Table 2:** Summary of graphene‐modified polymer FRPs (gPFRPs)

Classification^a)^		Type of filler [wt%]	Fiber type [volume fraction, %]	Dispersion [composite manufacturing method]	Reported mechanical properties	Reported electrical or thermal conductivity	Focus of the research	Ref.
Thermosets Thermosets	Epoxy	TrGO [0.3–0.5]	CF [58.7] and GF [55]	Three‐roll‐mill process Prepreg filament winding	Reduction of delamination area in CFRP by 7% and by 22% for 0.3 and 0.5 wt%, respectively	−	Improvement of compressive strength after impact through the use of TrGO	[125]
		GnPs [0.25–1.5]	CF	Mechanical mixing Prepreg	TS 51.82–66 MPa FS 96.54–115.46 MPa FM 2.22–2.63 GPa (+19%)	−	Investigation of mechanical properties of GnPs/epoxy reinforced composites	[121]
		GO [0.25–1.0]	T‐300 woven CF (WCF)	In situ polymerization of pyrrole or EDOT in the presence of GO Mechanical mixing followed by bath sonication Hand layup	–	6.5 × 10^−3^ (S cm^−1^) (0.5 wt% PPY/GO) 6.2 × 10^−3^ (S cm^−1^) (0.5 wt% PEDOT/GO)	Enhancement of electrical conductivity in C‐epoxy composites by integrating them with conducting polymers and GO	[128]
		GnPs (xGnP‐C‐300) [1–3]	Continuous unidirectional CF (UCF)	Mechanical mixing of GnPs and hardener Prepreg filament winding	GnPs fraction has a strong influence on the composite transverse tensile properties	−	Validation of multiscale modeling to determine the influence of GnPs volume fraction, epoxy crosslink density, and GnPs dispersion on the mechanical performance	[68]
		GO and GNPs [0.5]	UCF 12K	Three‐roll‐mill process Prepreg and lay‐up (16 plies)	Mode I fracture energy (*G*_Ic_) (kJ m^−2^) up to (+51%) 0.5 wt% GO 0.64 0.5 wt% GNPs 0.65	−	The increase of the interlaminar fracture toughness of CF composites enhanced with graphene nanospecies	[87]
		GnPs [1.0 vol%]	Weave CF	Ultrasonication of GnPs in solvent followed by mechanical mixing (400 rpm) Wet lay‐up and vacuum‐bagged (eight plies) Post‐curing	YM 48.6–51.0 GPa TS 601–639 MPa FM 58.1–68.0 GPa FS 586–679 MPa CM 9.0–11.9 GPa CS 288–387 MPa	Up to 0.55 (W K^−1^ m^−1^) (CFRP/GnP/SnW) (+40% relative to the unmodified laminate) (−55% in the through‐thickness of electrical volume resistivity (GnPs 1 vol%) Thermal conductivity up to +45%	Synergistic improvement in the through‐thickness thermal and electrical conductivity of the CFRPs by integration of graphene nanoplatelets or silver nanoparticles/nanowires	[123]
		GO [0.1–0.6]	T‐300 WCF	Ultrasonication of GO in solvent followed by mechanical stirring Prepreg and compression molding	FS 509–710 MPa (+66%) FM 28–35 GPa (+72%) Improvement in the interlaminar shear strength (ILSS) (for 0.3 wt% GO epoxy/CF composite compared to pure epoxy/CF composites) (+25%)	–	Improved mechanical properties of CFRP composites through the inclusion of GO	[69]
		PANI‐GO [15]	UCF 3K (CF/DVB)	Three‐roll‐mill process Hand lay‐up (12 plies)	YM 3.4 GPa ILSS from 15.6 to 27 MPa (+66% compared to CF/DVB‐PANI	3.5 × 10^−2^–6 × 10^−2^ (S cm^−1^)	Electrical conductivity and ILSS enhancement of CFRPs through synergetic effect between GO and polyaniline	[129]
		GnPs [0.1–0.4]	CF [0.05–0.25]	Ultrasonic dispersion of fillers followed by compression molding	Ultimate load 0.82 (kN) Maximum deformation 4.376 (mm) FS is enhanced by 35%	–	Investigation of the optimum percentage of graphene and CFs providing enhanced mechanical properties in polymer‐based composites	[130]
		GO [1.0–3.0]	GF	Mechanical mixing Hand layup	TS 343.27–407.06 MPa Elongation 10.53–15.84 mm FS 21.65–24.52 MPa Hardness 144.77–193.29 HRB	–	Investigation on Mechanical Properties of GO reinforced GFRP	[131]
		GO [0.1–0.]5	CF	Mechanical mixing followed by bath sonication Hand layup	TS 498.2–518 MPa YM ≈ 16–18 GPa FS 560–602 MPa FM 34.3 GPa (+14%)	–		[132]
		GO or rGO [0.05–0.4]	T300 UCF	Ultrasonic dispersion Hand lay‐up prepreg and compression molding	ILSS 105.80–117.45 MPa (+11%) interfacial shear strength (IFSS) of GO (+32%) IFSS rGO (+21%)	–	The effect of integration of graphene into CFRP on mechanical and EMI shielding properties of composites	[133]
		0	GF	Ultrasonication of GO in resin followed by mechanical stirring Hand layup	Cryogenic ILSS (+32.7%) @ 77K	–	Improvement of cryogenic ILSS of FRP epoxy‐based composites through the use of GO	[134]
		GO [2.0]	UCF	Ultrasonication of GO in DMF followed by centrifugal and mechanical mixing Hand lay‐up prepreg (24 plies)	Mode I fracture toughness (+170.8%) Fracture resistance (+108%)	–	Investigate the effects of GO interleaf on the fracture toughness of CFRP composites	[135]
		GO [0.1–0.5] Ethylenediamine (EDA): GO [0.1–0.5] Diaminodiphenyl sulfone (DDS): GO [0.1–0.5] *p*‐Phenylenediamine (PPD): GO [0.1–0.5]	T700S UCF [40]	Chemical functionalization of GO Mechanical mixing and ultrasonic dispersion Hand lay‐up and vacuum bagged infusion	TS 737 MPa GO (+22.4%) TS 831 MPa EDA:GO TS 747 MPa DDS:GO TS 850 MPa PPD:GO FS 1200 MPa GO (+76%) FS 900 MPa EDA:GO FS 1050 MPa DDS:GO FS ≈ 1400 MPa PPD:GO FM 64 GPa GO FM 66 GPa EDA:GO FM 65 GPa DDS:GO FM ≈ 62 GPa PPD:GO	–	Use of functionalized GO to improve the mechanical properties of the matrix of CFRPs	[136]
		Silane: GO [2.0]	UCF	Chemical functionalization of GO followed by dip‐coating onto CF surfaces Prepreg fabrication, hand lay‐up (16 plies) and autoclave processing	Bonding strength (up to +53%)	–	Enhancement of the interfacial bonding strength of CFRPs using silane functionalized GO	[137]
		GnPs [0.1–0.5] COOH:GnPs COOH/NH_2_:GnPs	CF	Mechanical mixing of GO and functionalized GO followed by sonication Drop casting of resin	IFSS 72.7–79.0 MPa GnPs IFSS 73.9–80.2 MPa COOH: GnPs (+28.5%) IFSS 73.9–82.4 MPa COOH/NH_2_:GnPs (+32.05%)	–	Investigation of the IFSS in CFRPs when modified with functionalized graphene	[138]
		GnPs [5–12] NH_2_:GnPs	GF	Dispersion of GnPs into resin using a two‐step method based on probe sonication Wet hand lay‐up followed by hot plates press	FS ≈ 550–720 MPa FM ≈ 100–115 MPa	In‐plane conductivity ≈10^−4^ S m^−1^	Evaluation of electrical and mechanical properties of functionalized GnPs epoxy‐based GFRP composites	[122]
		GnPs [1.7–10.7]	PAN‐based 3K CFs	Ultrasonication followed by dip‐coating of CFs Immersing coated fibers in Epoxy and casting	IFSS 35.8–51.8 MPa (+45% compared with noncoated CFs)	–	Incorporation of GnPs into a CF‐epoxy interphase to improve IFSS in comparison with noncoated CFs	[76]
		rGO [90.25]	12 µm E‐glass	Dispersed in acetone and centrifuged then added to monomer epoxy followed by polymerization	(Properties of single coated fibers) TS 2289 ± 380 MPa (+17%) Weibull modulus	(Properties of single coated fibers) 500–1000 kΩ	The effect of carbon‐based (graphene and carbon nanotube) coating on the mechanical, electrical, and barrier properties of GF composites	[124]
		GnPs [0.1–0.3]	BF (200 g m^−2^)	GnPs dispersion into epoxy followed by mechanical mixing, hand lay‐up procedures for impregnation of BFs (ten plies)	TS 223–240 MPa YM 13.34–15.92 GPa US 0.042–0.045% FS 220.56–273.91 MPa FM 5.77–8.11 GPa Flexural strain 0.028–0.034% Impact strength 105–83 (kJ m ^−2^) (−21%)	–	The influence of GnPs inclusion on mechanical properties of BF/epoxy composite laminates	[72]
		GnPs [0.1–0.5]	CFs	GnPs‐resin dispersion preparation using ultrasonication and mechanical mixing Spray coating of GnPs dispersion onto CFs followed by vacuum‐assisted resin infusion (VARI) process (eight plies)	ILSS (+24.5%) FS (+27.2%)	Thermal conductivity 0.54–0.84 W mK^−1^ (+55.6%)	Incorporation of GnPs into CFRPs to improve the mechanical properties and thermal conductivity	[139]
		rGO dispersion [0.05–0.4]	CF	Dispersion of rGO into epoxy through ultrasonication Impregnation of CFs with rGO/epoxy suspension VARTM process	ILSS 27.8–43 MPa Impact energy 3.058–3.555 (J) Impact toughness K_IC_ 22.8–27.3 (MPa m^1/2^)	–	Effects of hydrazine reduced GO on the interlaminar fracture toughness of CFRPs	[140]
		GnPs and NH_2_:GnPs [0.5]	E‐glass	Dispersion of graphene particles into epoxy followed by impregnation of GFs VARTM process (eight plies)	Mode I 42.75–56.62 N Mode II 511.08–747.07 N Mode III 24.10–24.92 N	–	Influence of GnPs on modes I, II and III interlaminar fracture toughness of FRPs	[141]
		GnPs [1.0]	3K CFs [54]	Dispersion of GnPs into epoxy through mechanical mixing, Hand lay‐up followed by VB (eight plies), compression molding process and cured at 177 °C for 2 h	Compressive Strength 608 MPa (+10%) Mode I fracture toughness 191–203 J m^−2^	Electrical conductivity 13.1 × 10^−4^ S m^−1^ (+132%) Thermal conductivity in the transverse direction 0.72 W mK^−1^ (+6%)	Improvement of electrical, thermal, and mechanical properties	[142]
		GnPs [0.1–1.0] GO [1.0–2.0] rGO [0.01–0.042]	Unidirectional E‐glass	Dispersion of nanoparticles into epoxy followed by VARTM process (eight plies)	–	Thermal conductivity improvement MWCNTs 0.3% (+8.8%) GnPs 1% (+12.6%) GO 2% (+8.2%) rGO 0.042% (+4.1%)		[56]
		PANI‐GO [15.0]	3K CFs	Preparation of DVB‐PANI‐GO matrix Impregnation of the suspension onto the CFs followed by curing under the hot‐press	YM 3.4 GPa ILSS (+76%)	Electrical conductivity up to 6.36 × 10^−2^ S cm^−1^ (+150%) PAni:GO [60:1]	Electrical conductivity and ILSS enhancement of CFRPs through synergetic effect between GO and PAni	[129]
	Bismaleimide	Maleic anhydride functionalized GO (MAH‐GO)	UCF T700SC	Chemical functionalization of GO followed by in situ polymerization Compression molding method	ILSS 24.4% FS 1.51 GP GO (+11%) FS 1.75 GPa MAH:GO (+28.7%) FM 84.63 GPa GO (+27.9) FM 98.87 GPa MAH:GO (+49.7%)	–	Use of maleic anhydride functionalized GO for improving the interfacial properties of CFRPs	[115]
	Polyester	CuO:GO (10× 10^−3^ to 120× 10^−3^ m):(0.1‐1.2 wt%)	T‐300 WCF	Microwave synthesis of CuO nanoparticles onto WCF Three‐roll‐mill process to mix resin and GO Vacuum‐assisted resin transfer molding (VARTM)	TS (+61.2%) YM (+57.5%) ILSS (+89.9%) Impact energy absorption capacity (+154.8%)	Interlaminar electrical resistive heating (+78.9%)	Study the interfacial resistive heating and mechanical properties of GO:CuO enhanced PES‐based CFRPs	[116]
Thermoplastics	Polyurethane	Graphene dispersion [5 mg mL^−1^]	GF	Dispersion of graphite in DMF using bath sonication Hand lay‐up and vacuum‐assisted resin transfer molding (six plies)	Ultimate strain (US) 2.4% TS 60 MPa	–	Fabrication of graphene‐integrated strain sensing laminate composites based on TPU for SHM applications	[126]
	Polyamide	GnPs	Short GF	Dispersion of graphene platelets and short GF within the polyamide PA6 matrix	TS ≈ 2.3 GPa US (1.0–2.0%) *G*_Ic_ 13 kJ m^−2^ CS up to ≈2.0 GPa Compressive strain 0.22%	–	Investigation of the crashworthiness response for graphene‐integrated hybrid polyamide‐based CFRP	[93]
	Polyamide	Master batch of graphene, with a 15% graphene content by wt [10.0–300]	UCF	Resin transfer infusion (RTI) process	*G*_Ic_ > 400 J m^−2^ Mode II fracture toughness (*G*_IIc_) < 2000 J m^−2^	Up to ≈0.0006 S cm^−1^	The effect of addition of graphite into PA12 on the electrical conductivity and fracture toughness of CF‐Epoxy composites	[127]

a) The reported increase is compared to the reference samples without graphene.

## 6 Performance Comparison of gPFRPs with Benchmark CFRPs

Research on gPFRPs to date has largely focused on theory and understanding the fundamentals of the materials processing and properties. Therefore, benchmarking gPFRPs as a novel material system in this field would enable us to gain an independent perspective on how well they perform compared to FRPs, which are already playing key roles in the market. CFRPs have become indispensable technical components of modern industries in recent decades and are being widely used for weight critical structures due to their excellent specific strength and stiffness, high corrosion resistance and good thermal dimensional stability. CFRPs are now identified as standard constituent materials in new aircraft structures. However, CFRPs exhibit anisotropic mechanical, electrical, and thermal properties which often make their design and application more complex compared to more conventional engineering materials. The growing demand for the application of CFRPs as the key component for structural solutions has necessitated the development of CFRPs with improved matrix properties to avoid the challenges associated with limited fracture toughness, heat dissipation and electric current flow paths within the composites.^[^ ^143^ ^]^ In this section, key properties of gPFRPs are categorized and compared by their performance characteristics against benchmark CFRP composites. In the absence of a specific regulation for graphene composites, it is hoped that this comprehensive comparison may provide a foundation for a focus on processes and standards for future investment in gPFRPs.

Some of the main material properties, as well as a number of considerations concerning processing and manufacturing of CFRPs and gPFRPs, are summarized in **Table** **3**. Generally speaking, mechanical properties of gPFRPs have shown significant improvement compared to those of CFRPs. CFRP is an anisotropic material, whose mechanical performances vary according to lamination angle and thickness. In addition to this, the mechanical performance of CFRPs is highly dependent on the fiber, matrix, and the interface between them. Also, the surface of pristine CFs is nonpolar, whereas the polymer matrix generally exhibits polar characteristics. The strength of the interfacial bonds between the CFs and matrix is, consequently, poor, thereby precluding achievement of the ideal mechanical properties of the composites. Embedding graphene particles into CFRPs will increase the surface functional groups and interfacial adhesion between the CFs and surrounding polymer matrix^[^ ^81^ ^]^ and, thus, results in extraordinary synergy in the mechanical properties. Within gPFRPs the stress can be transferred from the microscale to the nanoscale reinforcement, improving the ultimate mechanical and fatigue properties, as discussed previously. Saying that, it is essential to have a uniform dispersion of graphene in the polymer matrix to achieve this improvement. TS is heavily affected by aggregation of the filler and that is why it is often found that there is a linear increase of the tensile modulus with increasing filler content, while TS saturates at lower filler percentages. Furthermore, GO is often preferred over few‐ or many‐layer GnPs (even though the modulus of GO is only about 25% of that of monolayer graphene) since the presence of functional groups in GO enables easier coupling interactions between the filler and the matrix. It has been shown that embedding GNPs can restrain crevice growth in the graphene reinforced hybrid nanocomposites and prevent the expansion of these cracks, which reduces the effects of delamination, minimizes fractures, and enhances the overall lifespan of CFRP composite laminates. Adding a low percentage of graphene into the polymer matrix will improve the mechanical properties of CFRPs, with at least 60% increase in TS, 100% increase in toughness and 45% increased elongation to failure.^[^ ^121^ ^]^

**Table 3:** Comparison of properties of established CFRPs with gPFRPs

Material systems Properties	CFRPs	gPFRPs
Stiffness	Higher stiffness compared with general symmetric material and saves 20–50% on weight^[^ ^149^ ^]^	Specific stiffness exceeds that of CFRPs due to the CF reinforcement as well as a strong synergistic effect through modification of carbon‐based filler with graphene.
Strength	Extremely strong (1900–3400 MPa)^[^ ^151^ ^]^ and lightweight material–4.5 times stronger and stiffer than any metal component in the industry for a given weight of material.	Significantly improved ultimate strength because the stress can be transferred from the microscale to the nanoscale reinforcement.
Toughness	Low fracture toughness due to the brittleness of the main matrix and weak interfacial bonds between the CFs and the matrix.	Fiber transfer of stress increases toughness without compromising strength or stiffness performance.
Wear	Typically, resistant to wear; wear performance depends on its application and loading conditions.	Exhibit very high resistance to wear.^[^ ^152^ ^]^
Fatigue	Known to have high fatigue strength. However, show a significant directional degradation of the stiffness and strength during cyclic loading. Thus, fatigue damage is initiated in various failure modes.^[^ ^153^ ^]^	Adding graphene dispersions into the fiber reinforced hybrid composite increases fatigue life.^[^ ^121^ ^]^
Conductivity	The high fiber orientation in CFRPs gives rise to materials with higher electrical conductivity levels than those found for particulate composites.	Addition of graphene into the polymer matrices increased the conductivity relative to that of CFRP composites.^[^ ^152^ ^]^
Corrosion	Excellent corrosion resistance.	Corrosion‐free
Thermal stability	Higher temperature tolerance compared to that of metallic components.	Stable at extreme working temperatures.
Manufacturing complexity	The main bottlenecks are the manufacture of the preform for RTM, and the actual injection and cure of the part. RTM is also a relatively slow method for large‐scale productions.	A crucial step will be the dispersion of graphene nanofillers to achieve a homogenous system.
Machining of final part	Reduced machinability in areas of fiber reinforcement.	Better machinability compared to that of CFRPs.
Implementation capability	Established manufacturing technologies are available	Minor modification to existing manufacturing route required
Raw material costs	Expensive raw material costs compared to metals.	GO or rGO dispersions which are typically used to fabricate GFRPs are inexpensive. For example, GO in solution sells for 99 Euros per 250 mL from Graphenea.^[^ ^154^ ^]^
Manufacturing and processing costs	No autoclave required (room temperature) and low pressures which lower the tooling cost. The tooling cost for compression molding SMC parts can be 40–60% lower than that for stamping metal parts.	Processing more difficult (and thus a bit costlier) than CFRPs.
Technology readiness level (on a scale of 10)	9	3
Manufacturing readiness level (on a scale of 10)	10	5

Typically, CFRPs are resistant to wear, demonstrating the potential to save significant amounts of money in the maintenance and repair of buildings and infrastructure. Savings of between 30–40% over service life are possible, mainly due to the inherent superior qualities of CFRP composites. However, they show significant directional degradation of stiffness and strength during cyclic loading in contrast to isotropic engineering materials like metals. gPFRP composites have shown increased fatigue life compared to those of CFRPs.^[^ ^121^, ^144^, ^145^, ^146^ ^]^ As with the positive influence of GNPs on the mechanical and toughening properties of nanoreinforced polymers and composites, the incorporation of graphene nanomaterials into the matrix of fiber reinforced epoxy laminates has contributed to significant improvements in the fracture characteristics and damage tolerance of FRPs.^[^ ^121^ ^]^

CFRPs are poorer electrical and thermal conductors than metallic alloys that have been used in aircraft and aerospace construction as principle materials. Electromagnetic fields can penetrate through less electrical conducting CFRP regions into the airplane. Without additional design features CFRP structures are susceptible to severe damage in the event of a lightning strike. The slow rate of passage of electricity through CFRPs leads to heat generation within the composite by a resistive heating process followed by a tremendous rise in temperature at the strike area causing resin pyrolysis and fiber damage.^[^ ^147^ ^]^ Inclusion of graphene nanomaterials into the polymer matrix, to develop electrically conductive matrix composites, can significantly increase the through‐plane electrical conductivity compared to that of CFRP materials used in the construction of fuselages. This would hopefully negate the need to utilize copper mesh which is employed for the prevention of damage caused from lightning strikes.^[^ ^123^ ^]^

CFRPs are also known to demonstrate good corrosion resistance.^[^ ^147^ ^]^ Carbon–carbon covalent bonds are extremely strong and naturally resistant to oxidation due to the bond strength (carbon is open to oxidation above 450 °C). Encasing these bonds within another polymer matrix that is also resistant to corrosion, such as epoxy or polyethylene offers the composite even more resistance to oxidation. However, elevated environmental conditions such as temperature and humidity can have profound effects on most of the CFRPs. The effect of moisture at wide ranges of temperatures can lead to degradation of the mechanical properties of CFRPs, particularly at the matrix–fiber interface. While the CFs themselves are not affected by moisture diffusing into the material, the moisture plasticizes the polymer matrix. Further, CFRPs showed limited success in metal matrix composite applications due to the formation of metal carbides and subsequent corrosion effects. CRFPs can induce galvanic corrosion in attached aluminum structures whereby the CF acts as the cathode on account of its more noble potential relative to aluminium. Graphene is an excellent material for cathodes and is frequently used in place of Pt in electrochemical cells. Well‐dispersed GnPs embedded in a polymer matrix could prevent corrosion owing to the relatively high aspect ratio of graphene sheets which enhances the oxygen barrier property of the matrix. Chang and co‐workers reported the improved corrosion properties of epoxy/graphene composites compared to those of pure epoxy for cold‐roll steel materials.^[^ ^148^ ^]^ In this study, an increased contact angle of water droplets was noted on epoxy/graphene composite surfaces (≈127°) compared to that of pure epoxy (≈82°). The hydrophobic epoxy/graphene composites repel moisture and further reduce the water/corrosive media adsorption on the epoxy surface, protecting the underlying metals from corrosion attack.

Improved machinability can also be achieved through graphene functionalization of the matrix in CFRPs compared to traditional CFRPs. Damage‐free machining of polymer matrix fiber reinforced polymer composite materials with conventional machining methods such as drilling, cutting, milling, grinding, etc. is a highly challenging process even under proper conditions due to the anisotropic and nonhomogeneous structure of composites and to the high abrasiveness of their reinforcing constituents. Moreover, the drilling process becomes a challenging issue during assembly.^[^ ^149^ ^]^ Machining of CFRP composite materials was found to reduce the strength and fatigue life of the component. Graphene functionalization of CFRPs will increase the interfacial adhesion between the CFs and surrounding polymer matrix and, thus, decrease failures such as resin–fiber debonding and microcrack formation caused by machining and cutting.

**Figure 10:**
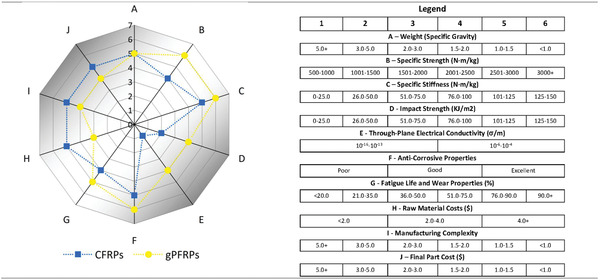
Radar charts comparing gPFRPs with benchmark CFRPs in relation to part performance and scalability potential. Each material system has been evaluated in terms of A) weight, B) specific strength, C) specific stiffness, D) impact strength, E) through‐plane electrical conductivity, F) anti‐corrosive properties, G) fatigue life/wear properties, H) raw material costs, I) manufacturing complexity, J) final part cost; a low value corresponds to manufacturing complexity, higher cost of raw materials, and manufacturing.

High prices have so far limited the use of CFRPs for high‐volume applications while GO or rGO dispersions, which are typically used in the fabrication of gPFRPs, can be inexpensive due to the very small amounts of nanoparticles used to achieve high performance. Estimates show that improvements in the production process over the next few years should cut carbon composite costs, significantly (up to 50%).^[^ ^150^ ^]^ Added to this, more processing steps are involved in the manufacturing of gPFRPs compared to CFRPs. Tooling can range from very low‐cost to high‐cost, life‐long molds. RTM is one of the typical methods used to manufacture CFRPs. This is usually done at room temperature (no autoclave required) under low pressures which keep fabrication costs low. RTM takes advantage of the broadest range of tooling (either hard or soft tooling) for any of the composite processes, depending upon the expected duration of a particular manufacturing run. Soft tooling involves the use of either PES or epoxy molds, while hard tooling may utilize cast machined aluminum, electroformed nickel shell, or machined steel molds. Tooling costs for other manufacturing methods, such as compression molding, can be 40–60% lower than that of stamping metal parts. In comparison, while development of gPFRPs is achievable through current available manufacturing technologies (relatively high manufacturing readiness level (MRL)), the final product costs may be higher compared to CFRPs due to additional processing steps. On the downside, the research on gPFRP composites is still at a low TRL. Significant gaps of knowledge and technology remain to be filled before laboratory achievements can be transferred to pilot plant or industrial‐scale production. Industrial demand is a critical driving force behind the direction and development of this related knowledge and technology. This includes increasing efficiency of manufacturing scalability and reproducibility, extending current material models and forming new regulations on materials specifications and characteristics. A brief comparison of CFRP and gPFRP properties is given in Table 3 and **Figure** **10**.

## 7 Fiber‐Treated FRPs

Regardless of the improvement in many physical properties, the reduced flowability of the polymer matrix after the incorporation of GnPs would restrict the processing of FRP composites. Higher temperatures and pressures are usually required during processing which pose a more critical demand on the equipment required. Furthermore, GnPs may risk being filtered out by the microscale fibers leading to an uneven filler distribution, affecting the overall properties of the composites. An alternative strategy introduced more recently is to dope graphene onto the surface of the fibers used for reinforcement which results in a 3D effect between plies under loading.^[^ ^31^, ^155^ ^]^ Subsequent incorporation of already‐prepared “smart fabrics” into insulating polymer matrices to produce gFFRPs will not only enhance their mechanical performance significantly but also create other functionalities such as in‐built sensing, monitoring, energy storage, and information processing.

The prevention of fatigue and corrosion failure in structural parts has been a critical concern in aircraft engineering for many years.^[^ ^156^ ^]^ Composite processes permit manufacturers to produce fewer, lighter and more complex parts, consolidating previously separate metal components. Moreover, the damage tolerance and high strength‐to‐weight ratio of composites has motivated designers to expand the application of these advanced materials in structural parts.^[^ ^2^ ^]^ This practice, however, further complicates inspection.^[^ ^126^ ^]^ Though well‐established procedures exist to detect the presence of structural fatigue, unanticipated failures of aircraft and civil structures still occur and have compelled the engineering community to take a fresh look at current designs and reassess the effects of structural aging. As a result, the aviation industry has recognized the need for real‐time structural health monitoring (SHM) systems and more innovative ways to deploy them in situations where complex structural geometries create access limitations. Current SHM approaches include embedding some type of fiber Bragg grating sensors within the composite or applying a metallic sensor to the outside surface of the structure.^[^ ^157^ ^]^ Smart gFFRP structures employ in situ sensors that are incorporated into the structure for real‐time cure monitoring coupled with a nondestructive inspection (NDI) technology. This permits greater access to difficult‐to‐inspect areas of complex structures, eliminating the need for disassembly. This continuous monitoring of structural parts will considerably increase their operational safety. The information acquired in real‐time would also benefit the understanding on fracture mechanics of composites, building confidence in their use and broadening their applications.

Among a number of monitoring parameters, strain is the most useful indicator of structural response to external loads for identifying damage. Localized changes include cracks, impact damage and delamination of materials.^[^ ^158^, ^159^ ^]^ In structural composite systems, strain‐gauge sensor‐based metallic alloys and optical fibers are two of the most extensively investigated techniques for SHM purposes. Metallic strain gauge sensors use a single‐sided evaluation technique for assessing the microstructural condition and distributed damage state of the material. On the downside, conventional metallic strain gauges suffer from low gauge factor (G‐F—a fundamental parameter of the strain gauge is its sensitivity to strain, expressed quantitatively as the G‐F, (G‐F = (∆*R*/*R*)/ε, where ∆*R*/*R* is the normalized resistance and ε is the mechanical strain) and fixed direction strain sensing.^[^ ^158^ ^]^ The integration and adaptation of small actuation and sensing elements such as piezoelectrics, micro/nanoectromechanical systems (MEMS/NEMS) into and onto the structures including polymer matrix composites, metal matrix composites, ceramic matrix composites, and even monolithic metallic materials have allowed the reliable observation of cracks in structures from a size of 5 mm upward. While this method has been proven to effectively work on laboratory scaled specimens, as for most SHM technologies, proof of concept in real structures under in‐service operational conditions is still lacking.^[^ ^160^ ^]^ Optical fiber sensors are also widely used in health monitoring of CFRP composite structures, offering (tensile or compressive) strain and temperature readings.^[^ ^161^ ^]^ In recent years, the focus has changed to packing and mounting Bragg grating arrays for SHM of large composite structures. This resulted in a simple and inexpensive vacuum‐assisted infusion method for the application and bonding of optical fiber sensor networks to large structures. The study showed that sensors can be bonded over large distances, making a large‐scale fiber optical sensor network feasible.^[^ ^162^ ^]^ Optical fiber sensors are also hindered by costly and susceptible failure due to defects in composite laminate structures. Moreover, practical issues like deployment of the optical fiber to the structure and connectors need to be addressed. Also, the timely detection of damage occurring at the subsurface, such as microcracks, before they can propagate and become distributed over large areas, is of critical importance.^[^ ^163^ ^]^

The piezoresistive nature of graphene (along with its other unique properties including high selectivity, fast response, and recovery times) allows for its use as a sensing element within a composite structure. In particular, the large surface area and planar geometry of graphene, as well as its excellent conductivity with small bandgap, would allow the fabrication of gas/chemical vapor, electromechanical, pH, mass, electrochemical, and optical sensors.^[^ ^164^, ^165^, ^166^ ^]^ Nonconductive fibers serve as strong scaffolds in gFFRPs which is compatible with composite manufacturing processes to secure graphene to a specific orientation and location. Additionally, and possibly more importantly, the scaffold allows the material to be handled in a dry state. It is also believed that the complexity of the conductive network helps increase the response of the sensor to damage. The high surface area of graphene on the fiber and the mechanical strength of the fiber both help to ensure that the sensor is intimately connected with the bulk composite. It has been suggested that a grid or matrix of sensing elements in a row and column array could use the resistance of each sensor node or crosspoint to more accurately locate damaged regions.^[^ ^167^ ^]^ Based on these facts, gFFRPs can perform a self‐sensing function as the bulk conductivity of the composite changes as it undergoes damage. This method is also expected to generate the most manifest effect on the fiber–matrix interface, modifying interlaminar fracture toughness, hardness, delamination resistance, etc.^[^ ^157^ ^]^ From this, early‐state graphene‐based sensors have shown many advantages over conventional sensors that include being smaller, lighter, stronger, easier to use and fabricate and less expensive. GFFRPs are expected to match the sensitivity of conventional strain gauges while enabling for easier integration into composite structures (since the sensor itself is a composite) allowing the potential of sensing over large areas and in previously inaccessible locations.

### 7.1 Processing Techniques for Fabrication of Graphene–Fiber Assemblies

Development of graphene‐treated fiber sensors has shown great progress over the past decade as an interdisciplinary field that applies the principles of engineering and materials science to the development of smart composites. This approach provides a great deal of control and flexibility, allowing for fine adjustments to the concentration and distribution of graphene on and within the textile, as well as its basic structure. It would also allow an extremely high number of sensing elements to be embedded within a composite structure with minimal cost, weight, or performance penalty. This technique can also be extended by assembling repeating alternative patterns of graphene‐substrate on top of each other to create multilayered hybrid sandwich structures.^[^ ^168^, ^169^ ^]^ A number of coating methods have been used to produce graphene‐treated fibers including spin‐, dip‐ or spray‐coating, electrospraying, and electrophoretic deposition (EPD).^[^ ^170^ ^]^

**Figure 11:**
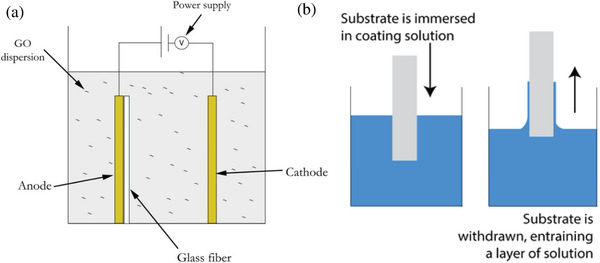
Schematic diagram of a) EPD, Reproduced with permission.^[^ ^174^ ^]^ Copyright 2019, Elsevier and b) dip‐coating process.^[^ ^175^ ^]^

EPD is a versatile cost‐effective material processing technique that continues to gain interest as a method for the deposition of graphene on different substrates. In this method, as shown schematically in **Figure** **11**a, an electrical potential is applied to electrodes immersed in a dispersion of charged nanoparticles. The stability of the suspension, its concentration, the size of the suspended species and the applied voltage affect the quality of the resulting deposits. Both aqueous and nonaqueous suspensions have been used for the EPD of graphene onto CF‐ and GF‐continuous fibers, woven fabrics and chopped fibers.^[^ ^171^, ^172^, ^173^ ^]^ However, the most common strategy reported is the use of GO as the starting material in the suspension. A pristine graphene is hydrophobic and cannot be dispersed uniformly in most aqueous solvents. GO is negatively charged in solution with high mobility and its oxygen‐functional groups are hydrophilic. Therefore, an aqueous suspension of GO is preferable as a starting point for EDP.^[^ ^155^ ^]^ The quality of the GO coating on the surface of CF can be guaranteed by using EPD, producing a coating with a uniform and controllable thickness. Surfactants (charging additives) are used in many cases to provide high zeta potential and electrophoretic mobility to the suspended particles as well as keeping the suspension stable.

Dip‐coating and spray‐coating are also convenient ways to assemble graphene materials onto textile substrates (see Figure 11b). Dip‐coating was found to be a suitable approach to coat nanofillers onto the surface of GF as the fiber is a thermally unstable and electrically insulating material, incompatible to CVD and EPD.^[^ ^155^ ^]^ However, these techniques often result in nonuniform film thicknesses on the substrates due to aggregation of GO/rGO sheets. Spin‐coating can overcome this issue as the thickness of the coating layer can be tuned by varying the concentration of the graphene dispersion which can result in minimal wrinkling.

Several woven and braided fabric structures based on nylon,^[^ ^176^ ^]^ polyester,^[^ ^177^ ^]^ cotton,^[^ ^178^ ^]^ carbon,^[^ ^155^, ^179^ ^]^ glass,^[^ ^180^ ^]^ and other textile materials^[^ ^181^, ^182^, ^183^ ^]^ have been made electroconductive using conductive inks including suspensions of graphene‐based materials. Their choice as the substrate for applying graphene inks is mainly based on their nonconductivity, strength and compatibility with composite manufacturing processes. Coated continuous fibers or fiber fabrics can be either used as they are or chopped down to short fibers for processes such as or melt compounding. Graphene‐assembled fibers have been used in thermoplastics and thermoset composites.^[^ ^66^, ^73^, ^85^, ^174^, ^184^, ^185^, ^186^, ^187^, ^188^, ^189^, ^190^, ^191^ ^]^ However, at present, the majority of reported gFFRPs rely on the application of thermoset resins.

### 7.2 Current Achievements in the Fabrication of gFFRPs

The development of smart gFFRPs has proven to be a promising area of research to meet the ever‐increasing demand for in situ real‐time SHM systems. Graphene‐based inks (GO, rGO, or GnPs dispersed in a solvent) have been used to coat CF‐ and GF‐continuous fibers, woven fabrics, and chopped fibers, using a variety of coating methods including, but not limited to, EPD, dip‐coating, spray‐coating, electrospraying, and spin‐coating. Graphene‐doped fibers have been reported for use in strain monitoring, pressure sensing and fluid flow monitoring applications.^[^ ^192^, ^193^, ^194^ ^]^ Graphene‐coated textiles have also been used lately as smart geotextiles (known also as geofabrics) for many structural applications.^[^ ^181^, ^195^ ^]^ Imagine IM Pty. Ltd., an Australian‐based company, in cooperation with Geofabrics Australasia, launched its graphene coating assembly line in 2016, initiating the production of a world‐first conductive geotextile.^[^ ^196^ ^]^ gFFRPs are created through the sequential embedding of layers of graphene‐doped fabrics into an insulating polymer matrix. As is observed for gPFRPs, most of the recent studies regarding preparation of gFFRPs have been focused on the use of thermoset matrices, in particular epoxy.

**Figure 12:**
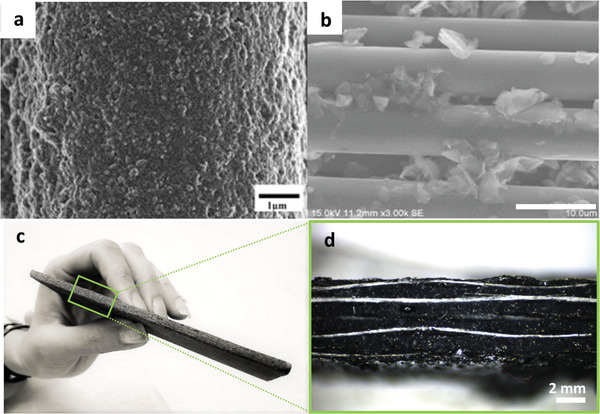
SEM micrograph of a) GnP‐coated CFs. Reproduced with permission.^[^ ^88^ ^]^ Copyright 2019, Elsevier. b) GnPs‐coated glass fabric. Reproduced with permission.^[^ ^198^ ^]^ Copyright 2020, Elsevier. c) Multilayered gFFRP composite, and d) Optical photograph of the cross‐section of gFFRP composite.

GO can be dispersed in fiber sizing agents and then coated onto the surface of fibers by pulling the fibers through or dipping the fibers into the modified sizing agent. Zhang et al. have reported improved interfacial shear strength (IFSS) and interlaminar shear strength (ILSS) properties (by up to 70.9% and 12.7%, respectively) for epoxy composites with the coated CFs compared to a composite with desized CF.^[^ ^197^ ^]^ Surface wettability of CFs by epoxy resin was noticeably improved by the deposition of GO on the fibers which increases their surface free energy. Additionally, the TS and modulus of GO‐coated CF‐reinforced epoxy composites were also higher than those of normal composites.^[^ ^197^ ^]^ Qin et al. manufactured GnP‐coated CFs/epoxy composites using a prepreg and lay‐up method which resulted in 52%, 7%, and 19% increases in 90, 0°FS and ILSS, respectively, in comparison to noncoated CFs/epoxy composites. Meanwhile, incorporating GnPs in the CF/epoxy interphase improved the electrical conductivity by up to 7 × 10^−2^ S cm^−1^ through the thickness direction by creating a conductive path between the fibers.^[^ ^88^ ^]^ Furthermore, Moriche et al. have recently described the electrical conductivity and strain monitoring capability of composites reinforced by GFs, dip‐coated by nonfunctionalized and –NH_2_ functionalized GnPs (see **Figure** **12**b).^[^ ^198^ ^]^ The resulting composite contained nonfunctionalized GnPs with an electrical conductivity of (9 ± 7) × 10^−5^ S m^−1^. In contrast, when NH_2_‐functionalized GnPs were used, the nanoparticles adapted to the surface of the fibers resulting in a major improvement in the electrical network created along the fibers, yielding an electrical conductivity in the order of 10^−2^ S m^−1^. The created electrical network conferred piezoresistive properties to the GF fabric. The sensitivity values, obtained under tensile loads, were reported to reach gauge factors between 840 and 16 400. Ongoing research continues to produce better electrical performance, mainly for sensing applications. Our group have recently demonstrated the use of highly conductive aqueous graphene‐enabled inks to prepare multilayered reinforced composites for real‐time monitoring purposes (see Figure 12c,d).

Commercial products of long GF‐ and CF‐reinforced thermoplastics are available, for example, pre‐consolidated laminates (organo sheets) from SGL Carbon group—a leading manufacturer of products based on carbon‐based in Germany—where CFs and GFs are bonded with a thermoplastic matrix (PA6) with the aid of special sizing in the form of long fiber‐reinforced pellets, UD tape and woven sheets.^[^ ^31^ ^]^ Notable is the fact that limited cases describe the preparation of graphene modified FRP composites employing thermoplastic polymers. Larkin et al. reported the spray‐coating of graphene onto the surface of plasma‐treated CF and preparation of graphene‐modified poly‐ether‐ether‐ketone (PEEK)/CF laminates by hot‐pressing at elevated temperatures.^[^ ^199^ ^]^ In 2015, an injection molding approach was described for fabricating a GO‐coated CF‐reinforced Polyethersulfone (PESU) composite.^[^ ^66^ ^]^ It was shown that the GO coating on short carbon fiber (SCF) surfaces leads to an enhanced SCF/PES interfacial adhesion which resulted in improved tensile and flexural properties. Li et al. employed a similar composite manufacturing methodology to prepare GO‐coated SCF/PES composites and explored the effect of GO incorporation on the mechanical properties of as‐fabricated composites at a typical cryogenic temperature (77 K). GO‐coated SCF/PES composites displayed greatly enhanced cryogenic mechanical properties with the highest values compared to neat SCF/PES composites. A summary of gFFRP composite investigations is presented in **Table** **4**.

**Table 4:** Summary of graphene‐enhanced fiber‐treated reinforced polymers (gFFRPs)

Classification		Type of filler [wt%]	Fiber type [volume fraction, %]	Deposition [composite manufacturing method]	Reported mechanical properties	Reported electrical or thermal conductivity	Focus of the research	Ref.
Thermosets	Epoxy	Positively charged GO	GF	O_2_‐plasma treatment followed by layer‐by‐layer (LbL) assembly for the surface modification of a GF Dip‐coating in resin	IFSS 28.86 MPa	–	Enhancement of interfacial adhesion of fiber and matrix through surface modification of GFs by GO and Aramid Nanofiber (ANF)	[200]
		GnPs [1.0–4.0]	PAN‐based CFs	Treated by UV/ozone oxidation Coating CFs using stainless steel rollers (GnPs suspension in NMP) Prepreg using drum‐winder technique Hand lay‐up and autoclave processing	FS 1400 MPa 90°FS (+52%) 0°FS (+7%) FM 140 GPa ILSS ≈80 MPa (+19%)	7 × 10^−2^ (S cm^−1^) (increase of 165% compared to the epoxy‐only coated CFs)	Improvement of mechanical and electrical properties of CF composites with incorporation of graphene	[88]
		Multiwalled CNTs [0.05] GO [0.05]	GF	EPD method Dispersion of fibers in epoxy	The TS and failure strain of composites are 60.8 MPa and 4.2%, 59.9 MPa and 4.1% for CNT‐CFRPs and graphene‐GFRPs, respectively.	Graphene‐GF [7.9 ± 4.1 mΩ] CNT‐GF [7.4 ± 2.3 mΩ]	Monitoring temperature and strain changes in FRPs incorporating CNT and graphene materials	[186]
		GO [0.1–0.6]	GF	EPD process	Increase the fiber/matrix ILSS in GF reinforced epoxy composites (219%)	–	Improvement of ILSS between GF and epoxy matrix through treatment with graphene materials	[174]
		GnPs	CF	EPD process Epoxy prepreg through molding	ILSS up to 20.7–55.5 MPa FS 693.6–949.4 MPa FM 29.2–51.3 GPa Elongation 0.023–0.028 (mm mm^−1^)	Resistivity 25–75 × 10^3^ Ω cm	Investigation of electrical and mechanical properties of xGnP/Cu integrated CFRP composites	[187]
		rGO [0.02–0.06 g]	CF	Chemical grafting of GO on CFs (self‐assembled) Resin transfer molding (RTM)	–	Resistivity 1.16–1.46 Ω cm	Grafting GO onto CFs and its synergistic effect on shape memory properties of the composite	[188]
		Ag:GO [0.02–0.02 to 0.02‐0.06]	CF	Grafting Ag nanoparticles GO onto CFs followed by RTM	–	Resistivity 0.23–0.28 Ω cm (Ag:GO grafted CF mats)	Study the synergistic effect of CF and GO on the shape recovery performance of nanocomposites	[189]
		GO [0.5 mg mL^−1^]	CF	EPD process Hand lay‐up molding method (20 plies)	FS 285–324 MPa (+14%) ILSS 47–63 MPa (+34%)	–	Investigation of the mechanical properties of GO‐integrated CFRPs	[85]
		GO [0.25 g L^−1^]	PAN‐based 12K CF	EPD process Preforms fabrication followed by VARTM	ILSS 36.7–57.1 MPa (+55.6%)	–	Influence of surface properties of GO/CF on interfacial properties of fiber/matrix	[201]
		5 µm GO [1.5] 15 µm GO [1.5]	12K UCF	Sizing onto CFs through dip‐coating RTM	ILSS 47.50 MPa (+39.5%) ILSS 41.33 MPa (+21.8%)	–	Effect of the GO sheet size on mechanical properties of CFRP composites	[190]
		GO–NH2 [0.2]	T‐300 WCF	Chemical grafting of GO‐NH_2_ onto CF Prepreg fabrication	ILSS (+36.4%)	–	Directly grafting of functionalized GO	[202]
		GO [0.5] EDA:GO DDS:GO PPD:GO	Bi‐directional woven GF	Mechanical mixing and ultrasonic dispersion Hand lay‐up and VB (eight plies)	Mode‐I fracture toughness/GO (+27%) Mode‐I fracture toughness/PPD:GO (+69.5%) Mode‐I fracture toughness/DDS:GO (+70%) Mode‐I fracture toughness/EDA:GO (+93%)	–	Enhancement of the fracture toughness of epoxy‐based FRP composites through matrix modification with functionalized GO	[203]
		GO [0.2–1.0] Silane:GO [0.2–1.0]	T700S CF [50 to 55]	Chemical functionalization of GO followed by dip‐coating onto CF surfaces Hand lay‐up and vacuum bagged infusion	IFSS 64.8 MPa (Silane:GO [0.5 wt%]) (+60%) ILSS 83.46 MPa (Silane:GO [0.5 wt%]) (+19%) TS 1150 MPa (GO [0.5 wt%]) TS 1543 MPa (Silane:GO [0.5 wt%]) (+15%) YM 34.2 GPa (GO [0.5 wt%]) YM 48.7 GPa (Silane:GO [0.5 wt%]) (+20%) FM 57 GPa (GO [0.5 wt%]) FM 82 GPa (Silane:GO [0.5 wt%])(+16%)	–	Effect of involvement of GO and silane‐functionalized GO on the mechanical properties of CFRPs	[204]
		GO [0.1 mg mL^−1^]	T700S 12K CF	EPD coat of GO sheets onto CF followed by ultrasonic and heat treatment Drop casting of resin	TS (+34.58%) IFSS 46.8–79.5 MPa (+69.87%)	–	Improvement of TS and interfacial properties of matrix/fiber via integration of GO into CFRPs	[172]
		rGO [0.001]	CF	EPD coat of GO sheets onto CF followed by chemical reduction (HI) VARTM (14 plies)	ILSS (+14%)	–	Improve the interfacial properties and wettability of CF through coating of CF with graphene using EPD	[205]
		rGO [1 mg mL^−1^]	3D braided T700‐12K CF	rGO dispersion in acetone followed by dip‐coating of CF VARTM	Peak impact force (+11%) Impact strength (+19.3%)	Thermal conductivity 1.03 W mk^−1^ (+13%)	Evaluation of thermal and mechanical properties of graphene integrated CFRP composites	[206]
		GnPs [0.1–1.0]	S2 Weave GF	Dispersion of GnPs into solvent followed by dip‐coating onto GF VARTM	FS 325–440 MPa (+29%) Fracture toughness ≈1000–1250 J m^−2^ (+25%) Absorbed energy up to 7.86 × 10^1^ J	–	Improvement of interlaminar and mechanical properties of GFRP composites with GnPs	[207]
		GO [1.5 mg mL^−1^]	T700SC 12K CF [0.5]	EPD process Functionalized fabric was cut into 3–5 mm sections and dispersed in resin	–	720 S m^−1^ EMI SE of 37.6 dB (+47.6%)	Effect of EPD condition on the EMI shielding performance of rGO/CFRP composites	[173]
Thermoplastics	Polyethersulfone	GO [0–1.0]	SCF [12.5]	GO sizing onto SCFs Injection molding	TS 119.09 ± 0.54 MPa YM 7.92 ± 0.02 GPa FS 182.51 ± 1.51 MPa FM 6.47 ± 0.08 GPa	–	Enhancement of mechanical properties of GO‐coated SCF‐reinforced PES composites by GO coating	[66]
		GO [0.2–0.5]	SCF [12.5]	Dip coating of GO onto CFs Injection molding	Up to 223.3 MPa @77°K (−196.15 °C) Up to YM 12.9 GPa @77°K (−196.15 °C) Up to FS 415.0 MPa @77°K (−196.15 °C) Up to FM 11.5 GPa @77°K (−196.15 °C)	–	Enhancement of cryogenic mechanical properties of PES‐based CFRP composites by GO integration	[191]
	Polyester	GO [0.25–1.0]	CF [0.25–1.0]	Electrophoretic deposition method (EPD) Reduction of GO‐CF by chemical reduction followed by dispersion of fibers in UP resin	–	7.13 (S m^−1^) With 0.75% mass fraction of rGO‐CF, the shielding effectiveness of the composite reached 37.8 dB (16.3% increase than that of CF/UP composite)	The effect of integration of GO onto CF on EMI shielding properties of PES composites	[184]
	Polyurethane	GO [0.1–0.3]	CF [1.0]	EPD process Prepolymerization method followed by compression molding	TS 51.07 MPa (+16.9%) ILSS 33.54 MPa (+74.4%) YM (+28.1%)	–	Investigation of the integration of GO on mechanical properties of PU‐based CFRP composites	[185]
	Polyamide 6	GO [1.0 g L^−1^]	Short BFs	GO‐PDA‐coated BFs followed by preparing GOPDA–BF/PA6 composite samples using injection molding (co‐rotating twin‐screw extruder)	Wear rate of the GO–PDA–BF/PA6 composite (−51%) Impact strength (+13.6%) FS (+12.7%) FM (+11.1%)	–	The effect of the use of GO into BF polymer composites on the mechanical and tribological properties	[73]
		GnPs [0.1–1.0 g L^−1^]	G‐BF [20]	Mixing graphene into polyurethane followed by Preparation of graphene coated BFs Composites made using injection molding (co‐rotating twin‐screw extruder)	TS 101.8–1216.1 (+18.2%) YM 120.3–1506.9 MPa (+23.9%) FS 132.9–4002.3 MPa (+34%) FM 178.2–6404.8 MPa (+60%) Wear rate GR‐BF/PA6 (+42.3%)	–	Preparation and mechanical evaluation of BF‐reinforced composite	[208]

### 7.3 Comparison with Benchmark Metallic Alloy Sensors and Optical Fiber Composites

Existing sensing and SHM technologies in the composite industry include optical fibers or strain‐gauge sensor‐based metallic alloys which may be embedded within the composite structure or applied onto the exterior surface.^[^ ^157^ ^]^ As noted previously, both options have demonstrated performance and cost restrictions.^[^ ^163^ ^]^ Development of state‐of‐the‐art smart gFFRP technologies provide real‐time information about the internal stress state and structural integrity of composites and, thus, ensure predictive maintenance. This section provides a comparison of novel gFFRP technologies against two of the major players in the industry, optical fibers, and metallic‐based strain gauge sensors, as a guide toward the targeted TRLs required to facilitate their industrial adoption.

Some of the major gFFRP material properties and manufacturing challenges are summarized in **Table** **5**. Metallic sensors are known to provide high electrical conductivities (10^−8^ Ω resistivity). However, these sensors can be bulky, expensive and only able to detect localized damage.^[^ ^209^, ^210^ ^]^ Moreover, current metallic strain gauges often suffer from low gauge factor and fixed direction strain sensing. In contrast, optical fiber sensors can be bonded over large distances, making a large‐scale fiber optical sensor network feasible.^[^ ^162^ ^]^ However, the timely detection of damage occurring at the subsurface level, such as micro‐cracks before they can propagate and become distributed over large areas, is of critical importance.^[^ ^163^ ^]^ Graphene‐assembled fiber sensors exhibit higher sensitivities than conventional strain gauges and can be readily integrated into a structural laminate to provide sensing over large sections and in previously inaccessible locations as the sensor itself is a composite and the need for disassembly is eliminated.^[^ ^158^, ^159^ ^]^ The high fiber orientation in gFFRPs gives rise to materials with higher in‐plane electrical conductivity levels than those found for particulate composites. The high surface area and complexity of the graphene on the fiber surface also help to ensure that the sensor is intimately connected to the bulk composite enabling a sensitive response of the sensor to damage.

**Table 5:** Comparison of gFFRPs properties with benchmark metallic alloys and optical fibers

Material systems properties	Metallic alloys (MA)	Embedded optical fiber composites (OFC)	Graphene‐enhanced fiber‐treated reinforced polymers (gFFRPs)
Conductivity	High electrical conductivity (10^−8^ Ω resistivity)	N/A	Leveraging ballistic conductivity of coated graphene textiles can greatly enhance the conductivity of gFFRPs and their sensing potential^[^ ^158^, ^212^ ^]^
Stiffness	Unlike SHM systems conventional NDI systems, are independent of the aircraft, applied on the outside surface of structure	Despite having high stiffness, CFRPs display a significant anisotropy in their properties and their mechanical performances are varied according to lamination angle, thickness and direction of the applied loading	Flexible and robust materials; Using braided fabrics in the composite manufacturing, specific stiffness far exceeds that of CFRPs
Strength	N/A	Strong in the direction of fibers^[^ ^151^ ^]^	Strong 3D structure with high elongation and TS along with better shearing, twisting and bending performance
Toughness	N/A	Lack of ductility (linear‐elastic stress–strain response until failure)	Tough materials with high FS
Wear	Not used during operation—N/A	The durability and service life of FRP composites, depends on its purpose, environmental parameters, etc.	
Fatigue	Lower fatigue life compared to that of CFRPs	CFRPs are known to have high fatigue strength	No detrimental impact on fatigue life from addition of graphene. There are cases where an improvement is reported due to crack path deflection ability of graphene on the fiber surface
Corrosion	Not exposed to environmental accelerating conditions for prolonged periods	CFRPs demonstrate excellent corrosion resistance	Corrosion‐free
Temperature	Undergo a transition from ductile to brittle failure as the temperature decreases (0 °C), strength unaffected	Higher temperature tolerance compared to that of metallic components	Tolerant to extreme working conditions
Manufacturing complexity	Deploy them in situations where complex structural geometries create accessibility limitations	Deployment of the optical fiber into the composite structure and connectors needs to be addressed. Shrinkage of the matrix after curing is also a critical concern that may affect the optical fiber sensor properties	The possibility of making (near) net shaped preforms as well as the exceptional formability of fabrics which allows the formation of complex shapes
Machining of final part	Good machinability	Reduced machinability in areas of fiber reinforcement	N/A Only require embedded anchor points for assembly purposes
Implementation capability	Fully developed	Fully developed	Established manufacturing technologies are available
Raw material costs	Much lower than optical fiber sensors	Expensive raw material costs compared to that of metallic alloys	Lower costs compared to optical sensors. The large‐scale production of graphene would reduce the price further in the near future
Manufacturing and processing costs		No autoclave required (room temperature) and low pressures which lower the tooling costs	An inexpensive coating strategy and a low‐cost fabrication route such as RTM is needed
Technology readiness level (on a scale of 10)	9	9	4
Manufacturing readiness level (on a scale of 10)	10	10	7

Considering the composition, geometry, and mechanical features of composite materials, optical fiber sensors have material and dimensional compatibilities with FRPs and can be easily integrated into composite materials without altering their mechanical performance. Although the diameter of an optical fiber is typically much larger than that of a glass or carbon fiber used in a composite structure, their mechanical properties are very similar. However, there are limitations such as fiber brittleness, difficulty in handling and their weak interface strength with the surrounding matrix. Weaker interface bonding strength tends to improve the fracture toughness by resisting the propagation of cracks through the matrix and reduces the effectiveness of stress transfer. In order to get the desired performance, interfacial adhesion can be controlled by surface treatment, such as plasma treatment, fiber sizing and coating, electro‐discharge and dry or wet oxidation.^[^ ^153^ ^]^ The fracture toughness of carbon fiber reinforced plastics is governed by the following mechanisms: 1) debonding between the carbon fiber and polymer matrix, 2) fiber pull‐out and 3) delamination between the CFRP sheets. Fibers coated with graphene nanomaterials create an interphase in the polymer matrix which enhances the load transfer at the fiber matrix interface and, thus, the interfacial adhesion.^[^ ^157^ ^]^ This has a significant effect on the fiber–matrix interface, modifying interlaminar fracture toughness, hardness and delamination resistance. Moreover, 3D fabric structures, with high elongation and TS along with better shearing, twisting, and bending performance, improve mechanical properties in composites. It is worth noting that this comparison only considers loading in the direction of the fibers; mechanical properties in the transverse direction not being considered here.

The behavior of composites under fatigue loading is completely different to that of metals. Fatigue in metals occurs by the initiation of a single crack, which then propagates until catastrophic failure occurs. In contrast to metals, damage build up in composites is in a global rather than a more localized fashion. Common damage accumulation mechanisms for composites (fiber–matrix debonding, matrix cracking, delamination and fiber fracture) can occur independently or interactively depending on the material properties and testing conditions.^[^ ^153^ ^]^ Additionally, despite their high initial strength‐to‐weight ratio, a design limitation of CFRPs is their lack of a definable fatigue endurance limit. This, theoretically, means that stress cycle failure cannot be ruled out. In other words, while steel and many other structural metals and alloys do have estimable fatigue endurance limits, the complex failure modes of composites mean that the fatigue failure properties of CFRPs are difficult to predict and design. As a result, when using CFRPs in critical cyclic‐loading applications, engineers may need to design in considerable strength safety margins in order to provide suitable component reliability over its service life. No detrimental impact on fatigue life from addition of graphene into CFRPs is reported to the best knowledge of the author. Indeed, there are cases where an improvement is reported due to crack path deflection by the graphene on the fiber surface.^[^ ^144^ ^]^

One of the most significant advantages of composite materials is that composites can provide long‐term resistance to severe environmental conditions and wide temperature changes, while metallic sensors corrode easily once exposed to accelerating environmental conditions for a period of time. Despite this, embedded optical fiber composites (OFC) are susceptible to moisture absorption, and a non‐negligible loss of tensile stiffness and strength can occur with prolonged immersion in water as well as a reduction in IFSS. This is because absorbed water can modify the elastic–plastic behavior of the resin which can lead to decohesion of the matrix–fiber interface and, thus, composite performance degradation may occur during use. Chean et al. investigated the effect of water immersion on the interfacial adhesion of OFC with and without the inclusion of GF laminates.^[^ ^211^ ^]^ They found that increasing the GF content lowered the water damage effects at the polymer interface. For GF concentrations higher than 40%, a strong reinforcing effect was observed which is probably due to the reduction of polymer chain mobility in the presence of GFs. This would enhance the stiffness of the surrounding composite material, which, in turn, affects the stiffness of the optical fiber in the surrounding composite material.^[^ ^211^ ^]^ The corrosion and wear properties of gFFRPs are expected to outperform OFCs due to their improved fiber–matrix ILSS characteristics.

Metallic‐based sensors are seen as an inexpensive sensing approach but their deployment onto structures with complex structural geometries creates accessibility limitations. Optical fiber sensors are costly compared to that of metal sensors. The uniform integration of the optical fibers into the composite structures is an essential criterion which needs to be taken into account in order to achieve the required performance. For a successful installation a number of factors need to be considered, including

i) The fiber type used, in particular the coating type needs to be selected to match the temperatures and pressures of the molding process.
ii) The fiber needs to be positioned such that the risk of microbending is minimized.
iii) The ingress/egress points need to be engineered into the component.

**Figure 13:**
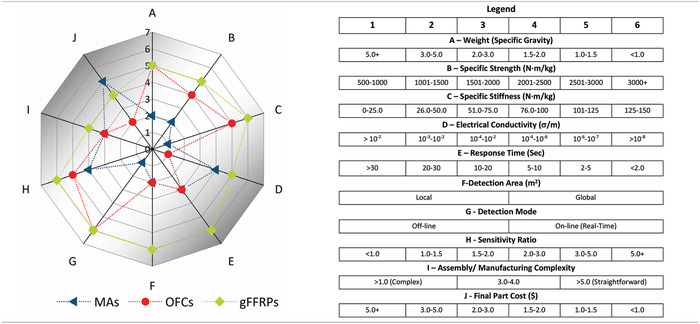
Radar charts comparing gPFRPs with benchmark MAs and OFCs in relation to part performance and scalability potential. Each material system has been evaluated in terms of A) weight, B) specific strength, C) specific longitudinal stiffness, D) electrical conductivity, E) response time, F) detection area, G) detection mode, H) sensitivity ratio, I) part assembly and manufacturing complexity, J) final part cost; a low value corresponds to manufacturing complexity and higher cost of raw materials and manufacturing.

In this way, a properly embedded sensor can be considered an integral part of the structure and will last the lifetime of the structure. gFFRPs are expected to match the sensitivity of OFCs, while enabling easier integration into composite structures and a cheaper fabrication route compared to that of OFCs. From this, gFFRPs provide lighter, stronger, easier to use and fabricate and less expensive SHM solutions over conventional sensing approaches. Yet, like many new technologies, this method has only been shown to work effectively at the laboratory scale. Proof of concept in real structures under in‐service operational conditions is an essential requirement in leveraging this technology and promoting TRL. **Table** **5** summarizes a comparison of gFFRP, OFC and metal sensor properties. **Figure** **13** also displays a comparison with benchmark MAs and OFCs.

## 8 Health and Environmental Considerations

The rapid advancements in the field of graphene materials have been accompanied by slower progress in the understanding of their impact on the environment and toxic effects in humans. Due to the increasing exploitation of graphene‐based materials, and the consequent discharge in the environment, their potential toxic impacts are becoming an urgent issue. Large‐scale production, leaching out from developed products, accidental spills during industrial production, and improper segregation and disposal of the derived wastes might result in significant release and accumulation of graphene‐based materials in the environment.^[^ ^213^ ^]^ Upon being released into waters, sediments, and soils, they may interact with a variety of physicochemical and biological factors, thus possibly causing considerable destructive effects to the environment with implications at the ecosystem level. It is, therefore, imperative to understand the interaction between the graphene materials and the environmental systems for predicting their risks.^[^ ^214^ ^]^

Humans can be also exposed to graphene by different routes, especially through inhalation, skin contact, and oral exposures during the synthesis of graphene or production of other graphene‐based materials systems. Compared with other carbon nanomaterials, such as carbon nanotubes and fullerenes, little is still known about their toxic effects in humans due to insufficient records. Some studies clearly showed no particular risks associated with the use of graphene‐related materials; other investigations, on the other hand, have proven that some types of graphene might become a health hazard.^[^ ^215^, ^216^ ^]^ Skin absorption and inhalation are considered as the most viable exposure routes, especially as dry powders by thermal exfoliation of graphite as well as during their exploitation and disposal. One major concern in the toxicity assessment of graphene research is that “graphene” is a generic term which refers to a family of materials including a variety of forms such as few‐layer graphene, graphene nanosheets, GO, rGO, and graphite. It has been indicated that the health risk of graphene materials strongly depend on their physical properties, shape, and surface functional groups.^[^ ^34^ ^]^ Generally, graphene materials with small size, sharp edges, and rough surfaces easily internalize into the cell as compared to larger, smooth forms. Also, the specific surface area and bending stiffness were found to be dependent on the number of layers and for biological molecules, the more layers of graphene mean lower adsorptive capacity.^[^ ^217^ ^]^ Graphene Flagship has made considerable efforts to analyze and evaluate the potential impact of graphene‐based materials on our health, as well as their environmental impact. A Recently, a “classification strategy” was proposed by them which considers three easy‐to‐measure and quantifiable material characteristics to assess the toxicity levels in different graphene materials including the average lateral dimension of the flakes, the number of layers, and the carbon‐to‐oxygen (C/O) atomic ratio.^[^ ^34^ ^]^ This framework may facilitate the comparison between studies performed in different laboratories and enable the assignment of specific physicochemical properties with the safety behavior of graphene‐based materials. In a recent article, the role of hydrophilicity, negative surface charge and colloidal stability of aqueous GO in its biodegradation process are emphasized.^[^ ^218^ ^]^

It is a complex process to illuminate the action mechanisms in the health domain that need to be taken forward due to the mutual effects of graphene layers and the relevant environmental or living systems. To fully evaluate the safety profile of graphene materials, these activities need to be continually focusing on a thorough exploration of the biological responses of graphene‐based materials together with creating specific guidelines and waste regulations, experimental protocols and establishing, if required, constraints for the safety of use. Moreover, attention needs to be given to the critical role of benchmarking toxicity results with the other existing carbon‐based materials technologies. The inclusion of graphene as a representative industrial nanomaterial in the nanomaterial repository of the European Commission's Joint Research Centre (JRC) repository and the creation of a specific reference library of well‐characterized graphene‐based materials will be also fundamental for benchmarking purposes in basic and regulatory research.^[^ ^219^ ^]^ It is believed that the connection between properties and safety profile of graphene‐based components is necessary to develop materials that can improve industry standards.

## 9 Concluding Remarks and Future Perspectives

The relative maturity of composites take‐up in a broad range of industrial sectors, such as aerospace and aeronautics, and the potential for significant growth in advancing markets, such as in the automotive, energy sectors, defense and civil infrastructure and the demand for lightweight yet high‐performance materials is the main initiative toward the development of novel ground‐breaking technologies. Continuous fiber‐reinforced composites with thermoplastic matrix resins (short‐glass‐fiber‐reinforced Durethan BKV30H2.0 polyamide (PA) 6) were lately reported for large‐scale production in automotive manufacturing.^[^ ^220^ ^]^ Another example is the recent demonstration of bridge decks made entirely out of FRP composites.^[^ ^221^ ^]^ This ever‐increasing demand for the application of FRP composites as the primary constituents for structural solutions has necessitated the development of FRPs with improved properties to avoid the challenges associated with limited fracture toughness, complicated inspection procedures, heat dissipation and electric current flow paths within the composites.^[^ ^143^ ^]^ Recently, there is a growing preference for the use of nanoscale reinforcement in composites for structural applications as compared to microscale fillers which leads to significant changes in the component properties.

Graphene nanomaterials have recently attracted intensive academic and industrial interest due to their unique combination of mechanical, thermal, and electrical properties.^[^ ^15^, ^16^, ^17^, ^18^ ^]^ Graphene‐based materials are suggested to bring disruptive solutions to the current industrial challenges related to energy generation and storage applications, industrial monitoring, surveillance, security, interactive electronics, communication, lab‐on‐chip, environmental monitoring, transportation, and automation. Two main routes were taken for the introduction of graphene into FRPs; nanoaugmentation wherein graphene nanomaterials are homogeneously dispersed into the matrix of the composite material, and nanoengineering in which pre‐organized graphene‐doped fibers are used to create laminate composites. The recent exploitation of gFRPs has not only led to dramatic improvements in their structural properties but also turned them into smart self‐sensing components capable of responding to the structural changes.^[^ ^222^ ^]^ This multifunctional nature of gFRPs is a critical consideration for several industrial sectors that require the development of new materials which combine enhanced mechanical, electrical, and thermal properties. The advancement of smart structures with real‐time damage sensing and monitoring capabilities enables higher benefit, lower maintenance and increased operational security for the end‐user, and contributes to improvements in the quality of life of society.

The widespread promising scientific results on the applications of graphene composites and the increasing necessity in the use of smart materials systems with improved properties have created a substantial demand on the engineering community to progress gFRPs from “lab‐scale” to “industrial‐scale” production. Despite numerous laboratory successes, the adoption of advanced gFRPs is hindered by significant challenges. Supply chain challenges including graphene material quality, inconsistencies in graphene material properties and high cost, coupled with a lack of clear standards for graphene materials and their dispersions, have hindered the uptake of graphene by different industries. Scattered knowledge about hazard identification and risk assessment, composite processing technologies, and computational models on graphene‐modified composites for prediction of the life‐span of composite structures also affect the lead time for technology adoption. Novel micromechanical models for graphene composites need to be promoted and verified with experimental studies for high‐performance structural applications which allows for further information to be analyzed at lower manufacturing costs.

While many limitations still exist, fabrication of smart graphene‐enable reinforced composites has proceeded quickly in recent years. Briggs automotive company (BAC), working alongside both Haydale and Pentaxia, has recently reported the development of the lightweight BAC Mono R body using graphene‐enhanced carbon composite materials.^[^ ^220^ ^]^ Prior to this, Elmarakbi et al. has described the fabrication of the world's first prototype graphene composite component for an automotive application—a car bumper.^[^ ^223^ ^]^ Imagine IM Pty. Ltd.—an Australian‐based company—in cooperation with Geofabrics Australasia has launched its assembly line of graphene coating in 2016 that enables the production of a world‐first conductive geotextile.^[^ ^196^ ^]^ The literature offers valuable reviews on graphene materials and applications, however, few of these articles link the obtained scientific data on properties of gFRPs to their industrial applications while also considering the technology and manufacturing readiness levels to speed up their market acceptance. This review article provides a critical overview of the essential steps toward the development of multiscale composite materials including the scalable synthesis of graphene materials together with the corresponding manufacturing methodologies for the effective incorporation of graphene materials into composite laminates as well as the latest results achieved for gFRPs. Lastly, proposed material systems are compared with some available benchmark technologies enabling the targeted TRLs required to facilitate their industrial use. The ultimate goal of this work is to reduce the time to market by filling the research gaps especially for high volume products with less costs and risk for companies and the environment.

## Conflict of Interest

The authors declare no conflict of interest.
